# Hypothesis-free phenotype prediction within a genetics-first framework

**DOI:** 10.1038/s41467-023-36634-6

**Published:** 2023-02-17

**Authors:** Chang Lu, Jan Zaucha, Rihab Gam, Hai Fang, Ben Smithers, Matt E. Oates, Miguel Bernabe-Rubio, James Williams, Natalie Zelenka, Arun Prasad Pandurangan, Himani Tandon, Hashem Shihab, Raju Kalaivani, Minkyung Sung, Adam J. Sardar, Bastian Greshake Tzovoras, Davide Danovi, Julian Gough

**Affiliations:** 1 MRC Laboratory of Molecular Biology, Cambridge Biomedical Campus, Francis Crick Avenue, Cambridge, CB2 0QH UK; 2 Department of Computer Science, University of Bristol, Bristol, BS8 1UB UK; 3 Shanghai Institute of Hematology, State Key Laboratory of Medical Genomics, National Research Centre for Translational Medicine at Shanghai, Ruijin Hospital affiliated to Shanghai Jiao Tong University School of Medicine, Shanghai, China; 4 Centre for Gene Therapy and Regenerative Medicine, King’s College London, Guy’s Hospital, Floor 28, Tower Wing, Great Maze Pond, London, SE1 9RT UK; 5 Université de Paris, INSERM U1284, Center for Research and Interdisciplinarity (CRI), Paris, France

**Keywords:** Computational biology and bioinformatics, Genetics

## Abstract

Cohort-wide sequencing studies have revealed that the largest category of variants is those deemed ‘rare’, even for the subset located in coding regions (99% of known coding variants are seen in less than 1% of the population. Associative methods give some understanding how rare genetic variants influence disease and organism-level phenotypes. But here we show that additional discoveries can be made through a knowledge-based approach using protein domains and ontologies (function and phenotype) that considers all coding variants regardless of allele frequency. We describe an ab initio, genetics-first method making molecular knowledge-based interpretations for exome-wide non-synonymous variants for phenotypes at the organism and cellular level. By using this reverse approach, we identify plausible genetic causes for developmental disorders that have eluded other established methods and present molecular hypotheses for the causal genetics of 40 phenotypes generated from a direct-to-consumer genotype cohort. This system offers a chance to extract further discovery from genetic data after standard tools have been applied.

## Abstract (web summary)

Systematically predicting phenotypes or disease risks based on the information of an individual’s genetic variation remains an unsolved challenge. Here, the authors develop a knowledge-based approach for performing and evaluating hypothesis-free phenotype prediction directly from a human genome.

## Introduction

**Fig. 1:**
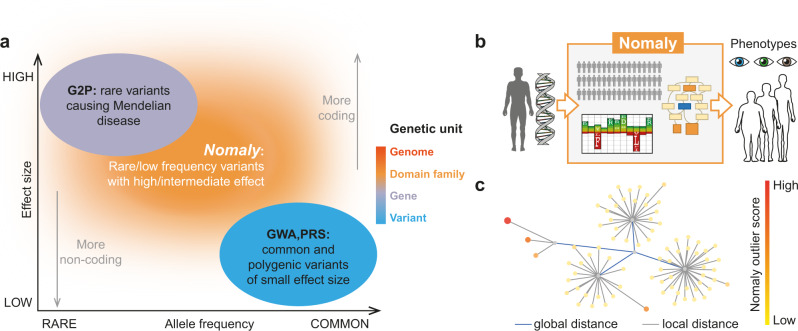
Framework incentive and design. **a** Positioning relative to heritability interpretation from two prevailing genetic association analyses^2^ of many. G2P: Gene-to-phenotype databases, GWA: Genome wide association, PRS: Polygenic risk scores. The colour bar shows the genetic unit of analysis employed by each method. **b** Framework overview. The method takes an individual’s genetic data as input and produces a list of ontology terms for which the person is a potential outlier. It uses a large background of genomes to which the individual is compared, ontology databases with gene-phenotype relationships, and evolutionary intolerance of mutations in protein domain families encoded by hidden Markov models (HMMs). **c** Schematic illustration of the genetic landscape for an ontology term (HP:0000834 ‘abnormality of the adrenal glands’) highlighting genomes with high outlier scores. Each node represents a genome, and edges are proportional to genetic distance in eigenspace – in essence, a reduced dimensional feature space between genomes.

Sequencing of human genomes holds great promise for using genetic information to guide medical discovery and therapy. And yet in general, advances in our ability to extract useful information from genetic data are not being made as rapidly as advances in our ability to generate the data, leading to a growing imbalance of effort. Systematically predicting potential organism-level phenotypes or disease risks based on the information of a person’s genetic variation remains an unsolved challenge. The influence of common variants on phenotypes can be quantified by statistical weights from genome-wide association studies (GWAS) and presented as polygenic risk scores (PRS)^1–3^, while effects of rare variants can be expressed in terms of intolerance to high-penetrance functional variants in the human population^4–6^ from both burden testing on common phenotypes and rare disease work in families. Nevertheless, these two leave a large area in the effect size – allele frequency space under-explored (Fig. 1a), more explicitly not accounting for the influence of a significant amount of non-synonymous uncommon variants of medium or low penetrance^2^. On one hand, rare or low-frequency functional variants are under-interpreted in GWAS due to the inherent difficulties in the statistical evaluation of rare events in population genetics^7, 8^. On the other hand, the statistical method for making gene-to-disease inference requires the support of multiple instances of high confidence predicted loss-of-function (pLoF) variants in a gene^4, 9^, and it has been shown that such instances are rarely observed. It is estimated that cohorts roughly 1000 times bigger than gnomAD (which contains 125,748 exomes) are needed to gather evidence of their existence in most genes^10^. On a few datasets with extensive broad phenotyping, Phenome Wide Association Studies (PheWAS)^11^ can leverage gene-based collapsing to address some rare variants. To further overcome these difficulties, and to apply to most datasets, we see the utility of a non-association-based method; ideally one incorporating the effects of rare or low-frequency variants, expanding our capability for discovery within the constraints of the existing scale of human genetics data.

Existing non-associative approaches that investigate genotype-to-phenotype relationships mainly use supervised network models, usually learned from large genomic variant databases focusing on specific phenotypes, e.g. antimicrobial resistance^12, 13^, yeast cellular phenotypes^14, 15^ and plant phenotypes^16^. These methods have demonstrated the possibility of using a knowledge-based strategy to make phenotypic prediction. However to achieve this, large datasets of millions of genetic variants (often through synthetic genetic arrays^17^) are needed and such datasets are only available for selected phenotypes, and such supervised models are not applicable to complex human phenotypes. We propose an unsupervised knowledge-based system (Nomaly), that makes ab initio predictions of potential phenotypes from thousands of ontology terms (Fig. 1b), leveraging the knowledge of protein domains through hidden Markov models^18–20^. Instead of being used to train a model at an early step, the phenotypes are used as a final step to evaluate which predictions performed significantly better than expected by chance. The underlying protein knowledge on which the ab initio models are based can be examined to provide molecular insights into the predicted phenotype, in other words unlike supervised models, the interpretability of our predictions is high.

**Fig. 2:**
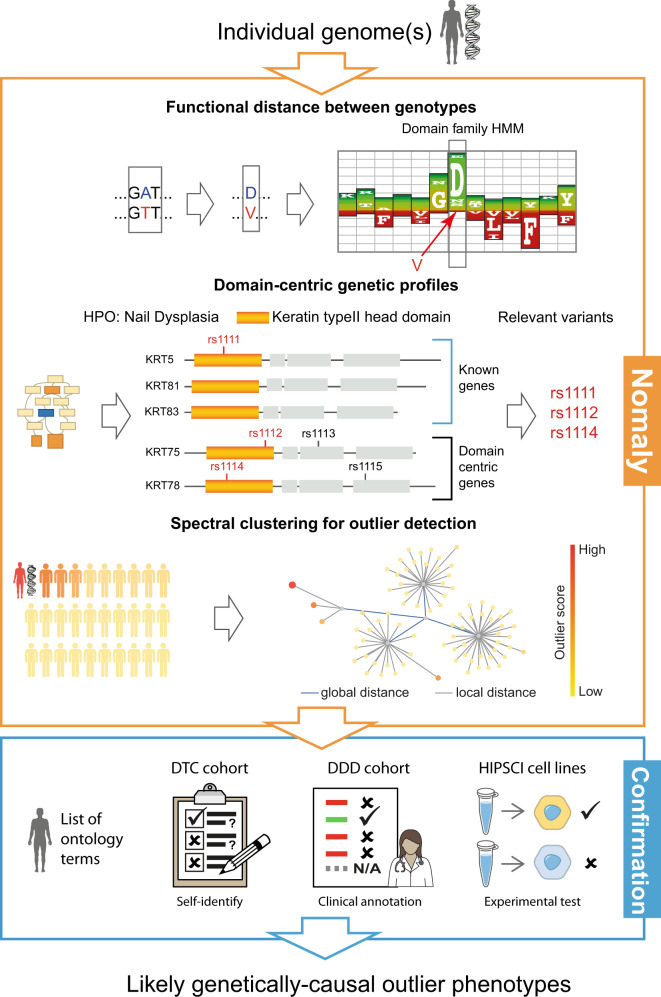
An outline of the genetics-first analysis framework. – see methods for detail. Genome data is inputted at the top and causal hypotheses are outputted at the bottom. In the orange top box (algorithm), firstly the functional distance between each missense variant is derived from domain-based HMM probabilities, scaled depending on zygosity (top row). Subsequently (second row) variants falling in the region of a gene with homology to an HMM representing a functional unit (domain), are collated into a genetic profile for a phenotype using domain-phenotype mappings inferred using dcGO^19^. This multi-domain collapsing of an ontology term can be likened to gene-based collapsing used in PheWAS^11^. Next (bottom row of the orange box) the profile of combined functional distances (from the top row) is used to calculate a genetic distance to every genome in the background. Spectral clustering of the distance matrix identifies which genomes are outliers under the profile (HP:0000834 in this illustration); nodes represent genomes and are coloured by outlier score (bottom right of the orange box). In the next (blue) box, only the top-scoring outlier phenotypes are passed to the confirmation stage, where some of these genetics-first predictions are identified as correct, giving a likely cause of the verified phenotype that was predicted.

The Nomaly system (Fig. 2) is built on the premise that a genetic extreme outlier can be defined that is predictive of an outlier in phenotype. Under this hypothesis, the system evaluates the genetic heterogeneity in the context of each phenotype. Consequently, not only is it able to consider the additive effect of multiple variants but also the non-additive combinatorial effect where some variants become relatively rare and deleterious in the presence of other more common variants. The challenging computation of this is made tractable via a linear algebra approach solving an eigenproblem (spectral clustering), described as segmentation-based object categorisation when used in image analysis^21^. A typical run includes a person or persons of interest and a large cohort-scale background, whereby outlier scores for thousands of terms in an ontology are calculated for each person of interest (Fig. 1b). The outlier scores represent the likelihood of being an extreme outlier in the ontology-specific genetic landscape with respect to the chosen background (Fig. 1c).

To evaluate performance, here we wish to systematically assess: (i) whether there is a global consistency in statistics between the actual phenotypes and predictions based on outlier scores; (ii) whether the predictive success rate can be quantified; and (iii) how novel the predictions are. In this work, we describe a knowledge-based framework and demonstrate its significance and usefulness by answering these questions using three independent datasets, namely: a cohort of 2248 participants specially recruited for this study (DTC, below), the well-established dataset of 1133 children in the Deciphering Development Disorders (DDD) study with the respective gene-to-phenotype database DDG2P that provided genetic diagnoses for 40% of these children (majority through de novo mutations)^6, 22, 23^, and the Human Induced Pluripotent Stem Cells Initiative (HipSci) stem-cell bank^24^ where there is the possibility to experimentally verify predictions on cellular phenotype.

## Results

### Evaluation of the predictive power with a direct-to-consumer genetics cohort (DTC)

**Fig. 3:**
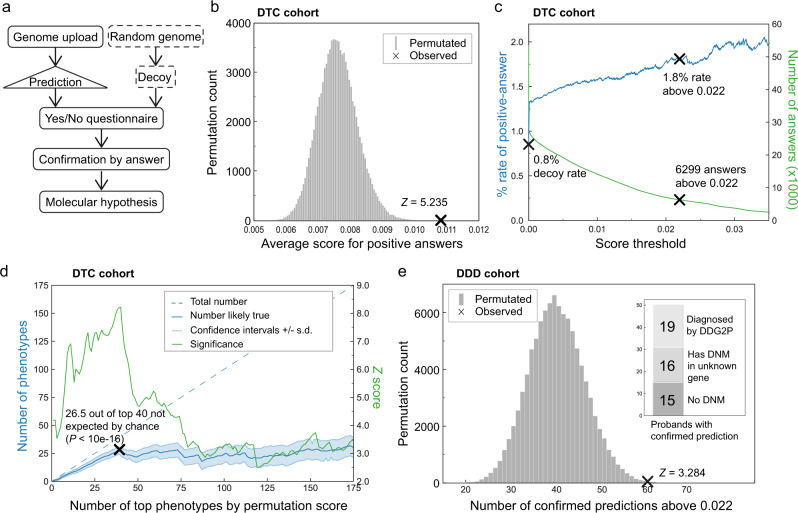
Evaluation of performance on DTC (a–d) and DDD (e) cohorts. **a** Participants upload their DTC genome data on which outlier phenotypes are predicted, then shuffled with outliers from a decoy genome randomly selected from the background, to create a uniquely personalised questionnaire. Answers are used to confirm true predictions against decoys. **b** A test of 100,000 random permutations of the dataset shows that observed scores are on average higher for confirmed phenotypes, with a *p*-value of 8.25e-8 against randomly permuted scores. **c** The rate of identifying confirmed phenotypes by score threshold (blue) and number of above threshold predictions (green); at the default threshold of 0.022 the rate is more than double the rate for decoys, with a *p*-value of 7.93e-7 against 100,000 permutations. **d** The significance (green) of the top phenotypes by within-phenotype permutation of answers 100,000 times, and (blue) for the top *x* phenotypes, the number left after subtracting from the total those expected by chance. *z*-scores were derived from testing the null hypothesis that similar results can be obtained if scores are assigned randomly (see methods). *p*-value is calculated from *z*-score in a right-tailed hypothesis test. **e** For DDD patients, the 60 above-threshold predictions confirmed by clinical annotation with a *p*-value of 5.12e-4 versus data from 100,000 random permutations, using the same hypothesis test procedure as in **d**. *Inset*: the 50 patients with top predictions compared to published data^22^ for whether a genetic diagnosis has been identified through DDG2P, and split by presence of de-novo mutation (DNM).

For the purpose of evaluation, we recruited a cohort of volunteers who had previously subscribed to direct-to-consumer (DTC) genotyping services (e.g. 23 and Me, AncestryDNA and others), or who were otherwise already in possession of personal genomic data files to participate in this study (Supplementary Fig. 1). To test the overall significance of outlier scores we presented each participant with a questionnaire asking them to self-identify, from a set of questions, any phenotypes from among 25 of their top-scoring ontology terms mixed equally with a further 25 top-scoring terms from a decoy – a randomly selected individual from the background (Fig. 3a). The DTC cohort generated 2248 questionnaires, yielding 94,966 yes/no answers across 3672 ontology terms and 2086 written comments; see methods for details of QC. Questions were intended to identify only outliers, so if the question design resulted in a high positive response rate (>5%), they were excluded for identifying a common phenotype instead of an outlier. By requiring participants to self-identify, the often-costly challenge of phenotype data collection is simplified, however, we sacrifice accuracy compared to expert assessment, introducing noise that will mask the true predictive power by an unknown amount.

A statistical permutation test of outlier scores versus positive self-identification by participants proves that the method is significantly predictive of phenotype at the <1% level (*p*-value: 8.25e-8, Fig. 3b). The average rate at which participants self-identify a random phenotype was measured as 0.8% by taking answers to decoy questions. However, participants identify at a higher rate for predicted phenotypes and this increases monotonically with score threshold choice (Fig. 3c). For high-scoring phenotypes the rate of self-identification is 1.8% (*p*-value: 7.93e-7), but with improved phenotype measurement this could be increased. These results show that the high-scoring phenotype predictions harbour statistically significant signals and that about one half (ratio of above-threshold positive answer rate to decoy rate) of the top-scoring predictions, verified through self-identification, are due to underlying genetic variation identified by the algorithm.

If a confirmed prediction has a genuine genetic basis and has not occurred by chance, then other predictions for the same phenotype in other participants are more likely to be true. This non-independence can be exploited and measured by per phenotype permutation tests. Further permuting across all phenotypes corrects for multiple hypotheses. Of the 40 phenotypes with the top statistic by permutation (Table 1 and Supplementary Data 1), only 13.5 are expected to have occurred by chance (Fig. 3d), which is a statistically much stronger result (*p*-value: <10e-16) than when considering predictions independently as above. Examples of a novel gene, recovery of a known variant, a novel variant in a gene related to a known gene, and mechanistic explanations are shown below.

**Table 1:** 10 representative examples of top DTC cohort phenotypes

Term ID Name (answers: yes/total)	Question
DO:DOID:896 Metal metabolism disorder (1/46)	Metal metabolism disorder is an inherited metabolic disorder that involves metabolic disturbances in the processing or distribution of dietary minerals. Has anyone in your family been diagnosed with metal metabolism disorder?
MP:0000286 Abnormal mitral valve (2/50)	Abnormal Mitral valve regurgitation is a backflow of blood caused by the failure of the heart’s mitral valve to close tightly. Have you been diagnosed with abnormal Mitral valve regurgitation through Echocardiography (ECG) or Electrocardiography (EKG) test?
MP:0010402 Ventricular septal defect (1/47)	The ventricular septum is the wall dividing the lower chambers of the heart. Have you undergone Echocardiography showing any defect in the ventricular septum?
GO:1901213 Regulation of transcription from RNA polymerase Il promoter involved in heart development (2/85)	Have you ever had magnetic resonance imaging (MRI) showing thickened heart muscle which may indicate cardiac hypertrophy (abnormal heart muscle enlargement) which is characterised by shortness of breath, general fatigue, fainting, and palpitations?
MP:0009445 Osteomalacia (2/96)	Osteomalacia is the softening of the bones caused by impaired bone metabolism primarily due to inadequate levels of available phosphate, calcium, and vitamin D, or because of resorption of calcium. Have you undergone an X-ray analysis showing the presence of osteomalacia?
HP:0000280 Coarse facial features (1/83)	Does your face seem to be coarse like to have large, bulging head, large lips and tongue, and small, widely spaced malformed teeth, etc?
HP:0002605 Hepatic necrosis (2/48)	Does your blood test show that you have elevated levels of liver enzymes which is the main symptom of hepatic necrosis or Have you been diagnosed with hepatic necrosis?
DO:DOID:11030 Corneal oedema (3/60)	Corneal oedema is the swelling of the cornea following ocular surgery, trauma, infection, inflammation as well as a secondary result of various ocular diseases. Have you been diagnosed with corneal oedema?
GO:0016447 Somatic recombination of immunoglobulin gene segments (1/60)	Have you or anyone in your family ever had blood test showing decreased levels of immunoglobulins which may indicate immunodeficiency-centromeric instability-facial anomalies syndrome (IF syndrome) (rare immune disorder), characterized by increased distance between bodily parts (hypertelorism), skin fold of the upper eyelid, unusually large tongue?
MESH:D010182 Pancreatic Diseases (2/77)	Have you been diagnosed with any pancreatic diseases like pancreatitis (pancreas inflammation), pancreatic cyst, cystic fibrosis etc?

Shown are the phenotype terms and corresponding questions presented to participants (out of 5,857 possible questions). The first column includes the term ID, name and in brackets the number of positive ‘yes’ answers out of the total number of questions answered by participants. For the complete list and more detail see Supplementary Data 1.

An assessment of potentially confounding factors (sex, ancestry and array type) establishes that the top phenotype predictions cannot be accounted for in this way. Although sex and three of the ancestry principal components (African, Gujerati Indians and Finnish) are predictive on the dataset, they are only significant (*p*-value < 0.05 at *Z*-score > 1.65) on 4 of the top 40 phenotypes. More importantly, none of the variables correlate (*r* > 0.1) with outlier score on any of the top phenotypes. As a baseline comparison to outlier scores, association statistics calculated on the dataset (Supplementary Fig. 1) did not reveal any significant variants due to the small cohort size of each phenotype. Aggregated association scores at high FDR subjected to a permutation test (Fig. 3d) identify 6(±2) out of a top 21 phenotypes having a significant variant association (Supplementary Data 5) not expected by chance at *Z*-score 2.9. The removal of points from the data for questions with outlier score >0.022 results in total loss of significance, implying that genetics-first selection of questions improved the power of the association results.

### Potential genetic diagnoses for Deciphering Developmental Disorders cohort children (DDD)

In addition to the evaluation on our own DTC cohort for significance, a comparison was made to state-of-the-art work in the field on the well-established DDD cohort for the purpose of assessing novelty. DDD consists of 1133 trios with developmental disorders who have been exome-sequenced and annotated with Human Phenotype Ontology (HPO)^25^ terms by clinicians ^6, 22, 23^.

Predictions on DDD were restricted only to HPO terms relevant to developmental disorders that were used for annotation by clinicians. Of these, 60 predictions above threshold matching clinical annotations for 50 patients were found (Fig. 3e). This rate is slightly lower, but consistent with the DTC results. Comparison to the published list of clinical diagnoses^6, 22^ shows that 62% (31) of these patients had received no genetic diagnosis, including 15 who have no de novo mutations (DNM); the published diagnoses in the DDD paper were made through DNM missense variants, inherited variants and rare chromosomal events in known genes using the developmental disorder gene-to-phenotype (DDG2P) database. Thus, plausible genetic explanations can be discovered for families not covered by the established G2P interpretation method (Supplementary Data 2).

A global analysis of the predictions made on DDD that match clinical annotations confirms significance of the method (*p*-value: 3.62e-4), although it is less than for the DTC cohort due to limited data, restricted to developmental disorder-specific HPO terms. Likewise, about 1/3 of the above-threshold predictions (*p*-value: 5.12e-4) are expected to be true instead of about 1/2 in DTC (subtracting decoy rate from prediction rate). Examples of a novel variant in a known gene and of a combinatorial effect are shown below. We conclude that, not only are the predictions significant on an independent dataset, but also largely non-redundant to those made by existing state-of-the-art methods, thus advancing the field.

### Application to interpreting genetic variants for cellular phenotypes on a large panel induced-pluripotent stem cell lines (HipSci)

Although HPO is the most common ontology used to annotate human phenotypes, and that used by DDD^22^, the DTC cohort study also included several other mammalian and disease ontologies (Supplementary Fig. 1b) and the gene ontology (GO)^26^. Despite being the ontology richest in data, GO performed worse on DTC than the other ontologies (*p*-value: 2.15e-2 for GO and *p*-value: 6.05e-7 for non-GO using threshold). This is presumably due to the difficulty in self-identifying molecular and cellular level terms, especially without recourse to invasive measurements on the person. The HipSci project provides exome sequence data for hundreds of iPS cell lines from different individuals, and thus offers an opportunity to examine this prediciton framework in the application to molecular and cellular phenotypes instead of patient-level phenotypes.

Outlier scores were generated for GO terms from the exome sequences corresponding to 427 HipSci samples, generating predictions potentially relevant to cellular phenotype. It is not possible to use this dataset to assess global performance versus phenotype identification as with the other two cohorts, but the top-scoring phenotype terms were explored to identify a prediction that could be empirically validated in vitro. The phenotype “negative regulation of centrosome duplication” within the “biological process” domain of GO presented high-scoring predictions (see below) for some cell lines, and was selected principally for its suitability for measurement in iPS cells with an existing assay.

**Fig. 4:**
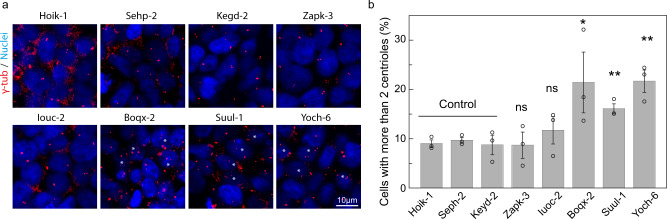
Experimental test for GO:0010826. This refers to 'any process that decreases the frequency, rate or extent of centrosome duplication'. **a** Representative examples of each of the indicated cell lines. Centrioles were detected by staining with gamma-tublin (γ-tub), nuclei were stained with DAPI. Asterisks indicate cells with more than two centrioles. The Hoik-1, Sehp-2 and Kegd-2 were control cell lines. **b** Histogram showing the percentage of cells with more than 2 centrioles per cell in the indicated cell lines. Results are summarised as the mean ± s.e.m. from 3 independent experiments (600-800 cells per cell line were analysed; each percentage from each experiment were shown as dots; *: *P* < 0.05; **: *P* < 0.005; ns: not significant). Specifically, the *p*-values are: Boqx-2 *P* = 0.045, Suul-1 *P* = 0.0021, Yoch-6 *P* = 0.0023 (one-sided t-test).

Centriole staining was carried out on HipSci cell lines corresponding to all five individuals predicted to be outliers from their exome sequence, and three controls not predicted to be outliers. Counting of the centrioles indicated that three out of five predicted cell lines displayed an elevated percentage of cells with more than two centrioles, suggesting defects in centriole regulation and cell cycle, confirming the accuracy of the prediction (Fig. 4). This phenotype would not be identifiable from symptoms in the DTC or DDD cohorts, but this empirical evidence demonstrates the value of the approach for discovery at the cellular or tissue level.

### Types of genetic outlier that explain potential phenotype outlier

**Fig. 5:**
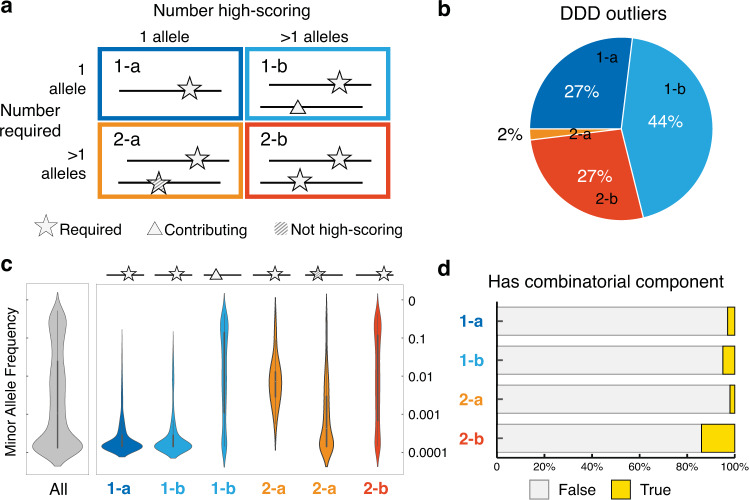
Types of genetic outlier. **a** Outliers classified by underlying genetic variants into 4 types: 1-a, single variant only required; 1-b, single variant plus contributing variant(s); 2-a, multiple variants but dominated by one high-scoring variant; or 2-b, multiple variants required. **b** Distribution of the four types of outliers in the DDD cohort. **c** Violin plots to show the distribution of log-scaled minor allele frequencies (MAF) of variants involved in all predicted outliers, and by type of outliers in the DDD cohort. Colours show different types of outliers, schemes show roles of the variants involved, similar in **a**. Specifically, all (grey): 3682 variants involved in at least one outlier prediction, 539 variants involved in type 1-a outlier (dark blue), 601 required (star) and 1548 contributing (triangle) in type 1-b (light blue), 416 required (star) and 20 contributing (triangle) in type 2-a (orange), and 1767 in type 2-b (red). Distributions were generated using a kernel density estimate in the seaborn package^47^. Boxes show quartiles and whiskers represent 1.5 multiple of interquartile range. **d** Percentage of outliers with a combinatorial component to the score with variants contributing non-independently.

**Fig. 6:**
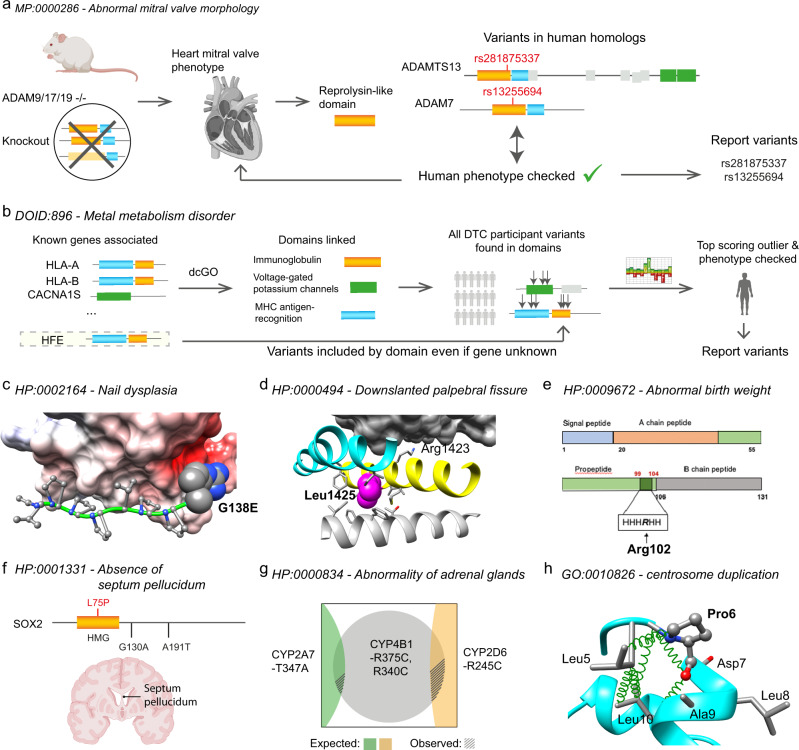
Examples. Any coordinates shown are relative to genome assembly GRCh37. **a** *Novel gene association*. ADAM7 [https://www.ncbi.nlm.nih.gov/gene/8756], ADAMTS13 [https://www.ncbi.nlm.nih.gov/gene/11093]. **b** *Known variant*. Chr6 Pos26093141, HFE-C282Y. **c** *Novel variant in related gene*. Chr12 Pos52913668, KRT5-G138E. **d** *Single variant*. Chr17 Pos29586054, NF1-L1425R. **e** *Single variant*. Chr19 Pos17927755, INSL3-R102C. **f** *Novel variant in known gene*. Chr3 Pos18143037, SOX2-L75P. **g** *Combinatorial effect*. 2 variants in CYP4B1-R375C/R340C and CYP2A7-T347A and CYP2D6-R245C. **h** *Experimentally validated on HipSci*. Chr3 Pos48414274, FBXW12-P6L. Panels (a) and (f) were created with BioRender.com.

An outlier in the genetic landscape of an ontology term, as detected by this approach, can be caused by a single or by multiple rare variants. In the case of multiple variants, each variant can be classified according to whether it is absolutely required to achieve a score above the threshold in the cohort, or whether it merely contributes to an above-threshold score; alternative variants can achieve above-threshold scores in different people (Fig. 5a, c). There can also be a combinatorial effect contributing to the score (e.g. Fig. 6g), whereby a variant becomes relatively rarer and more deleterious in the presence of a particular genotype consisting typically of a few common variants, exemplified here by having a higher outlier score if classified into a cluster (Fig. 1c) than if there is no cluster. The most common case (71%; Figs. 5b, 1-a/b) is where a single variant that is highly deleterious is sufficient to achieve the threshold although others may contribute additional score (44% cases; 1-b). Multiple variants being required to cause an outlier account for 29% of cases (2-a, 2-b). Combinatorial effects are important in a small minority of cases (Fig. 5d).

The majority (90%) of single-variant-based predictions were caused by a rare variant with minor allele frequency (MAF) <0.5% for a heterozygous genotype, or MAF < 1% for a homozygous genotype. However, not all rare variants result in a positive prediction, for example if it is not highly deleterious, or when many people from the chosen background harbour different rare variants for the phenotype, suggesting the ontology term is not highly evolutionary constrained. In multiple-variant-based positive predictions, 24% of variants are rare, and 55% are low-frequency (MAF < 1% heterozygous or MAF<5% homozygous) (Fig. 5c). Whilst our approach does not filter variants based on allele frequency (as illustrated in Fig. 1a), there is insufficient power to recover the effects of common variants given the size of the DDD or DTC cohorts. As each phenotype question is only presented to a small subset of participants, only effects of large magnitude emerge from rare genotypes.

### Examples

In Fig. 6 we show some examples of different types of discovery from the results. (1) Novel gene association. The representation in Fig. 6a shows how primary evidence from mouse knock-out experiments in genes ADAM9/17/19 led to a dcGO^18, 19^ link between a mitral valve phenotype and the reprolysin-like domain shared by these genes. Novel variants identified in respective domain of human genes (ADAM7, ADAMTS13) confirmed the mitral valve phenotype via questionnaire. (2) Known variant. In Fig. 6b a variant used to predict confirmed hemochromatosis in DTC participants was already known in ClinVar^27^. The diagram shows left to right: how multiple genes (including HFE) labelled with the ontology term were used by dcGO^18, 19^ to link it to domain families, then variants in DTC participants within these domains prioritised by HMM probabilities led to prediction. Had the HFE variant not been known (HFE not included as known), it is possible with this approach that it would have been discovered de novo by the DTC study on the basis of domain association. (3) Novel variant in related gene. The prediction in Fig. 6c includes other variants in keratin genes KRT75, KRT78 not previously linked to this ontology term. This variant is found in the head region (1–167) of the intermediate filament rod domain adjacent to an ELM motif involved in a protein-protein interaction with an SH3 domain. This figure shows a structural interpretation of the mutation of G138 to a polar negatively charged Glu disrupting binding to the electrostatic surface of SH3 domain from PDB structure 2GBQ.

Single variant. The single rare and functional variant (Fig. 6d) in the neurofibromin (NF1) gene is sufficient to produce the top-ranked score for this phenotype in DTC. The image shows a structural interpretation of the central domain of neurofibromin (with helices 6c and 7c in cyan and yellow respectively) bound to Ras (surface model, top) and the Leu residue at 1425 rendered as spheres. Substituting Arg for Leu at this position will disrupt the helical geometry and successful interaction with Ras. (4) Single variant. In Fig. 6e this single rare and functional variant in the insulin-like 3 (INSL3) gene is sufficient to produce the top-ranked score for this phenotype in DTC. The primary structure shows the variant lying within a conserved polar charged patch. The PDB structure 2H8B shows the two disulphide bonds that stabilise the structure. A mutation of the Arg to Cys could disrupt the polar charged region of the protein and even interfere with the correct formation of disulphide bonds. (5) Novel variant in known gene. Two independent cases in the literature of missense mutations in SOX2 (G130A and A191T) mean the example in Fig. 6f is already a known gene for the phenotype in the OMIM^28^ database. The phenotype was correctly predicted by our work on a DDD patient due to a novel variant in the HMG-box domain of this known protein. (6) Combinatorial effect. The phenotype in Fig. 6g that was correctly predicted on a DDD patient requires 2 variants in CYP4B1 (in linkage disequilibrium), plus additional variants. There is a combinatorial component to the score which raises this patient to the top rank in DDD for this phenotype. This Venn diagram shows that common variants are observed to co-occur (shaded areas) with the CYP4B1 variants much less than expected if the variants were independent (intersection areas). The top-ranked patient has all four variants. (7) Experimentally validated on HipSci. Figure 6h shows a homology model for F-box/WD repeat-containing protein 12 (FBXW12) using PDB structure 1NEX as template shows the proline at residue 6 that mutates to leucine in the variant, residing within the N-Proline box motif in N-terminal F-box domain. A proline at the N-cap position mediates hydrophobic interactions similarly to other N-cap residues Asn, Asp, Ser, and Thr. The mutation to leucine in this position impacts interactions within the motif, likely causing a change of specificity and/or affinity.

## Discussion

This paper describes a framework for performing and evaluating hypothesis-free phenotype prediction directly from a human genome. The key value is in providing potential genetic explanations for phenotypes that have been confirmed in the individual, which due to the high novelty and link to mechanism of the output, has potential for application to genetics-led drug target identification. Studying the combined effects on complex phenotype across variants in multiple genes is often impossible with simple statistical models, because of the lack of statistical power on existing cohort sizes. Also due to the lack of statistical power, rare or low-frequency variants are under-interpreted in GWAS^7, 8^. An ab initio model can partially overcome this limitation by testing a statistically small number of causally deduced direct predictions (a genetics-first approach). This knowledge-based framework with in silico and experimental validation approach described here has been shown to achieve this for exome missense variants. Our method, therefore, offers the ability to evaluate the effect of rarer genetic variants in a combinatorial way through linear algebra where statistical associative methods would not be applicable.

Hypothesis-free phenotype prediction with this genetics-first approach could be applied in principle to other ab initio models, but we chose to deploy a model based on protein domains, which are the functional units of proteins. Hidden Markov models built on protein domains^18^ enable the quantification of structural and functional effects of variants, and our domain-centric gene ontology (dcGO^19^) resource provides the link to phenotype. The domain-based model emphasises potential for novelty over coverage of known genes by carrying over the functional property of the domain across many genes.

Established genetic cohorts do not lend themselves well to testing a genetics-first approach since data is mostly only available for a restricted list of hypothesis-derived phenotypes – and usually not encoded in ontology terms, although increasingly attempts are being made to widen phenotype capture, e.g. using ICD-10^29^. The DDD cohort is one of the early large-scale studies to adopt HPO terms and was included in this analysis as a known reference point in the field. However, to truly test the genetics-first approach, we need an interactive cohort of participants. Participants were recruited for their willingness to provide their genotype data first, then respond to personalised phenotype data collection post-prediction. Evaluation on both cohorts similarly confirms the predictions as highly significant yet also characterises the predictions as having a high false-positive rate; expectations are that for roughly 1/3–1/2 of confirmed high-scoring predictions, the causal genetic explanations will be true. Combining results per phenotype is more powerful, showing that explanations for about 26 of the top 40 confirmed phenotypes are likely to be true (Fig. 2d). The predictions were also well-differentiated from results obtainable with other methods, confirmed by comparison to that achieved by DDD annotations of DNMs.

This method offers a powerful discovery tool for hypothesis generation from genetic data. The tool does not replace or compete with existing tools for human genetics which are largely aimed at being clinically actionable or offering effective intervention strategies. It rather complements and adds to them, aiming to enable medically high-value scientific discoveries first suggested from the analysis of genomic data. Independent validation of the hypothesis generated by the prediction with relevant assays is recommended. The expectation is that genes/variants will be mostly novel, and characterised by high magnitude of effect often from rare variants, sometimes severally, and occasionally acting combinatorially. In its essence, the tool is a genetic outlier detector, so it is important to consider the background with respect to which the individual is an outlier. In this work, we used the 1000 Genomes Project phase 3 (1KGP3) ^30, 31^ genomes as a diverse background with representation of all major ancestry in the DTC cohort, but by selecting a different background, the definition of an outlier can be adjusted to suit the desired research question.

The catalogue of high-scoring results from the analysis of the three cohorts: DTC, DDD and HipSci provide over a hundred putative causal genetic explanations for numerous developmental, cellular and human/mouse/disease phenotypes. In addition to the supplementary data tables, they are also provided online with an interface to aid browsing and searching the variants, their classification of type, genes, scores and phenotypes (https://supfam.org/nomaly), also including the database of 5,857 ontology questions as a resource for others.

The framework could also be used by other functional effect predictors, model organism ontologies and including known genes (to recover more of the less novel variants). We showed that even simple association statistics can be used within the framework, but that question selection is important, suggesting that a genetics-first step could be used to increase the power of PheWAS studies. Having proven principle on missense variants, we expect the future growth in this area by us and others will be by extension to other mutations e.g. indels and non-coding variants.

To sum up, the traditional approach to human genetics, where we ask “Does the data contain the answer to my question?”, has been turned on its head, and we instead ask: “For which questions does an answer lie within the data?”.

## Methods

### Ethics declarations

The DTC cohort study was granted ethics approval by the University of Bristol, via the Faculty of Engineering Research Ethics Committee with approval ID number 539500 (project ID 361 and amendment 2322). Informed consent from all participants was obtained. HipSci Lines samples were collected from consented research volunteer recruited from the NIHR Cambridge BioResource through (https://www.cambridgebioresource.org.uk). Initially, 250 normal samples were collected under ethics for iPSC derivation (REC Ref: 09/H0304/77, V2 04/01/2013), which require managed data access for all genetically identifying data, including genotypes, sequence and microarray data (‘managed access samples’). In parallel the hipsci consortium obtained new ethics approval for a revised consent (REC Ref: 09/H0304/77, V3 15/03/2013), under which all data, except from the Y chromosome from males, can be made openly available (Y chromosome data can be used to de-identify men by surname matching), and samples since October 2013 have been collected with this revised consent (‘open access samples’). The majority of samples were European. Work performed in the laboratories has been compliant with the Institutional Review Board directives for the experimental work and use of data.

### Nomaly framework

The framework consists of two primary parts: the predictive algorithm and the confirmation of predictions (Fig. 2). The algorithm takes an individual genetic data file (e.g. SNP array or exome) as input (top of Fig. 2, input to orange box) and outputs phenotypes for which the individual is predicted to be an outlier (from orange box into blue box). The definition of ‘outlier’ is made relative to a background comprising thousands of genomes (bottom left of orange box). For the DTC and HipSci cohorts, the 1KGP3 genomes were used as a background, but for individuals in the DDD cohort, the cohort itself serves as the background. There are thousands of potential phenotypes, taken from 17 biomedical ontology databases, each assigned a score (below) for how much of an outlier it is for the individual in question against the background. In principle any genome or any biomedical ontology database can be used for a given study. Hidden Markov models (HMMs) are used to estimate deleteriousness; models from the domain databases SUPERFAMILY^18^ and Pfam^32^ were used, but in principle any HMMs can be used or any other measure of deleteriousness from a variant effect predictor.

The confirmation step (blue box in Fig. 2) is the assessment of whether an individual is indeed an outlier for a phenotype suggested by the genetics-first analysis. In principle the assessment can be carried out in any way that lends evidence to confirm the outlier (the DDD study used at least two certified clinical geneticists to perform assessment^22^), but in this work automated matching of ontology terms was used. Predictions from the first step that are subsequently confirmed in this step become candidate hypotheses (output of blue box) linking a phenotype to variants via protein domains in genes.

Outlier scores (orange box in Fig. 2). The predictive algorithm includes (1) quantification of consequence of missense variants using evolutionary intolerance to the amino acid substitution in a protein domain, done by taking the difference in amino acid emission probability (analogous concept to FATHMM^33^) and (2) generating variant lists for ontology terms through domain-centric linking to phenotypes (taken from dcGO)^18, 19^. The mapping from variant to amino acids in proteins for (1) is done using the Variant Effect Predictor (VEP) tool^34^ (N.B. VEP used only for genomic mapping and no other functionality or scores are used) and mapping to domains using HMM sequence matching. The mapping of domains to ontology terms in (2) is combined with the variants falling within them from (1) to give a list of variants, commonly across multiple proteins, for each ontology term. Each ontology term is processed independently.

For a given term, a total genetic distance may be calculated between two individuals by summing the individual distances (defined as the log odds ratio of HMM probabilities for the two amino acids from the Dirichlet mixture) for all variants in the list for which they differ, depending on zygosity; distances are increased fourfold when both are homozygous and opposite. The all-against-all distances between the members of the background and the individual, and between each other, can be used to construct a distance matrix. This matrix is then translated to a similarity matrix through a Gaussian kernel and used as the input to spectral clustering to determine whether there is hidden structure in the genetic landscape of that term, namely by identifying the biggest gap in eigenvalues^21^. If no hidden structure is found, then the individual’s outlier score will be equivalent to the average Euclidean distance to members of the background; in this case individuals with very rare and highly deleterious genotypes will have a high outlier score (Fig. 5c). If a hidden structure is found by spectral clustering, K-means is then performed on the reduced-dimensional space derived from the top eigenvectors selected by the elbow method. In this scenario the outlier score becomes the sum of the local distance (from the cluster) and the global distance (between clusters) (Fig. 1c). To normalise for cluster size the global distance is multiplied by μ where:

1 $$\mu=\frac{{e}^{\frac{{size}_{{cohort}}-{size}_{{cluster}}}{{size}_{{cohort}}}\gamma }-1}{{e}^{\gamma }-1}$$

where *γ* specifies the penalty strength for large clusters; it was set to 9 in this study, conferring >99% penalty for large clusters with over 50% of the entire cohort.

Finally, since phenotypes have very different score distributions, a transformation (first described in Zaucha et al^35^) is employed to generate a universal score function comparable between phenotype terms.

2 $${s}_{{trans}}(p)=^3{\sqrt {\frac{{e}^{-\left(1+\tfrac{{s}_{{rank}}(p)}{N}\right)\varphi }}{{e}^{-\varphi }}\cdot \frac{s(p)}{\mathop{\sum}\limits_{p}s(p)}\cdot \frac{s(p)-{{{{{\rm{min }}}}}}\,s(p)}{{{{{{\rm{max }}}}}}\,s(p)-{{{{{\rm{min }}}}}}\,s(p)}}}$$

where *s*_*trans*_ (*p*) is the transformed score of participant p in a given term, *s*(*p*) is the original score and *s*_*rank*_ (*p*) is the rank of the original score within the Term. *φ* determines the strength of contribution of the rank to the total term score; it was set to 150 in this study, conferring the top ~2% a significant contribution.

### Combinatorial component

The non-linear method of clustering used can result in one or more variants being markedly *rarer* within one cluster relative to the entire background after genomes with other shared genotypes are grouped together. Thus a genome with a high global score due to the variants common to the cluster, and a high local score due to a variant rare only to that cluster, will have a higher total score than it would from its average Euclidian distance from the background. This additional score from variant rarity only increases in the presence of other specific variants, and is defined as the combinatorial contribution (as in Fig. 5d).

### Computational cost

Calculating spectral clustering requires a large computation, even when using low-level linear algebra BLAS^36^ libraries run on many threads in parallel. This is because it involves solving eigenproblems of a large matrix, which do not scale linearly with matrix size. For guidance, 5,800 terms on a batch of 50 SNP array genotype files with 2,504 background genomes (2554 x 2554 matrix) takes 21 hours with 12 threads (9.5 hours with 48 threads). Solving ~2,000 terms (independent eigenproblems) on a 3600x3600 matrix of exomes (including background) takes 3 hours using 600 threads.

### DTC cohort

#### Recruitment

Participants with access to personal direct-to-consumer genotype data were recruited anonymously online. Some participants were recruited in collaboration with OpenSNP^37^ and (separately) Sano Genetics. In this cohort 2,248 participations were recorded, with the participant website accessed from all over the world (Supplementary Fig. 1a). DTC genome data uploaded by participants was processed into a homogeneous format and quality-controlled with GenomePrep^38^, which also detects the sequencing method/genotyping array version. Imputation was not used for the analysis presented in this paper, although the main result of Fig. 3 was replicated on data imputed from the DTC genotype data (Supplementary Fig. 3) to confirm that there was little difference – only 4 terms outside the top 50 entered the top 40, with another 4 in the top 50 making a minor change moving up to the top 40. The IMPUTE2^39^ package was used with help from VCFtools^40^ and BCFtools by SAMtools^41^. A similarity matrix of all genomes in the cohort was calculated, consanguineous relationships were recorded and genetic duplicates removed; people submitting independent files from multiple providers were only allowed to participate once (Supplementary Fig. 1c–e). To prevent ‘gaming’ the study, for each participant a genome from the background was randomly selected, so the 25 ontology terms with the highest outlier scores for the participant could be randomly mixed with the 25 top-scoring ontology terms predicted for the background decoy genome to generate a personalised questionnaire with 50 questions. Each participant was invited to give a binary ‘yes’ or ‘no’ answer to whether they self-identify each phenotype, with the option to leave a comment (Supplementary Fig. 1b). In a small number of cases participants were recalled and invited to provide information supporting their answers for phenotypes of interest.

#### Process

The 1KGP3 genomes were used as the background. Before recruitment, the binary yes/no questions were designed with the aim of identifying outlier phenotypes for 5,857 ontology terms, including terms from the Gene Ontology (GO)^26^, Human Phenotype Ontology (HPO)^25^, Disease Ontology (DO)^42^, Medical Subject Headings (MeSH)^43^, and Mammalian Phenotype (MP)^44^ ontology databases (e.g. in Table 1). See Supplementary Data 3 at https://supfam.org/nomaly for an interactive version and database of ontology term to phenotype question mappings. Participant genome files were processed continuously in batches (eliminating within-batch relatedness) giving an approximately 4–12 hour turnaround between submission of file to the generation of personalized questionnaire; results can be influenced by other genomes in the batch which effectively becomes part of the background for each other. This is because all parts of the similarity matrix interact with each other during the solution of the eigenproblem.

#### Evaluation

We started with questions designed manually for 5857 different ontology terms. Not all questions were answered but in the end, 94,966 binary self-identified answers were received for 3672 questions, of which 1408 questions received at least one positive answer. Although questions were designed for participants only to self-identify when they are phenotypic outliers, often this was not achieved. To illustrate, a hypothetical bad question is “Do you have myopia?” whereas a good question would be “Do you have myopia worse than -6 dioptres”. Therefore, per-phenotype analysis was carried out for 342 ontology terms whose rate of participants answering ‘yes’ is non-zero and below 5%, and at least a total of 20 answers were recorded per term.

#### Permutation tests

Statistical evaluation in Fig. 2 was carried out using permutation tests with 100,000 iterations randomly re-allocating the outlier scores, testing the null hypothesis that similar results can be obtained if scores are assigned randomly. For panel b, the average outlier score given to questions that received positive answers (about 0.011) is compared to the averages from random permutations of the dataset. In panel d of Fig. 2, permuting the scores for each phenotype separately, yields a *p*-value on the sum of observed scores matching positive answers for each phenotype. All phenotypes can now be ranked by *p*-value (along the *x*-axis) for how well the observed scores predict the answers for that phenotype. The observed scores are then randomly permuted and all phenotype *p*-values recalculated; repeating 100,000 times gives a mean and standard deviation for the number of phenotypes expected by chance at any given *p*-value. At each point on the *x*-axis, subtracting the number of phenotypes expected by chance from the observed top phenotypes (*x*) gives the blue line – with confidence intervals. I.e. the number of phenotypes (*y*) likely to be true out of the top *x* phenotypes ranked by how well outlier scores match the answers.

### DDD cohort

#### Data source

Exome data from the DDD 1133 trio sequencing VCF files (accession code: EGAD00001001355), 1133 trio family relations, phenotype datasets, validated DNMs (EGAD00001001413), were obtained from the European Genome-phenome Archive at the European Bioinformatics Institute (EGA, https://ega-archive.org/).

#### Process

We ran the 1133 DDD probands, together with 1KGP3 genomes as background, using HPO as the ontology database. Only variants that pass all filters (as specified in the meta-data information from EGA downloads) are included. HPO terms that were used to clinically describe phenotypes in the 1133 trio were mapped to HPO terms in the v1.2 database used in our predictions. Due to the nature of the method, running all of the probands at the same time, in one distance matrix, means that the background is a mixture of 1KGP3 genomes and the other probands. If there was a common genetic cause shared by many probands, it would not get a high outlier score, but we assume enough genetic causes of the disorders are sufficiently independent to be eligible for detection.

#### Evaluation

For the DDD cohort, evaluation was automated through direct comparison, for each patient, of whether a high-scoring HPO term closely matches a respective clinical annotation. Defining a close match between ontology terms is challenging because the distance over the ontology graph varies wildly in biological meaning; adjacent terms can be very similar or very different. To define closeness, a measure of information content through the graph is needed, for which we used the cumulative number of patients annotated by terms traversing the graph as a metric. The close matches between HPO terms are listed in Supplementary Data 4. For example we found the following 4 terms sufficiently similar to define as close to each other: HP:0004097 ‘Deviation of finger’ (2 probands), super-term HP:0009484 ‘Deviation of the hand or of fingers of the hand’ (1 proband), and sub-terms HP:0009179 and HP:0004209 – ‘deviation’ and ‘clinodactyly’ of the 5th finger (56 and 2 probands respectively).

It should be noted that the evaluation assumes all potential HPO terms in the DDD database are considered by clinicians, and that an HPO term is *not* true for the patient if it is not annotated. This represents an underestimation of true phenotypes, but is a necessary assumption for a fair and automatic evaluation.

#### Comparison to published diagnosis by DDG2P

For patients with true positive predictions, we checked against the list of diagnostic variants in DDG2P from the initial publication^6^ and the list of validated DNMs (obtained from EGA) to see if there is a genetic diagnosis and if there is a potential uninterpreted DNM. The 1133 trio DNM list showed that at least one DNM was found for 738 (65%) of children (excluding synonymous, intron, and intergenic DNMs).

### HipSci cohort

#### Data source

HipSci exome sequencing data for healthy and diseased people (EGAD00001003514, EGAD00001003521, EGAD00001003522, EGAD00001003524, EGAD00001003525, EGAD00001003516, EGAD00001003526, EGAD00001003527, EGAD00001003517, EGAD00001003161, EGAD00001003518, EGAD00001003519, EGAD00001003520) were obtained from EGA. HipSci open-access exome-sequencing data were downloaded directly from the hosting website (https://www.hipsci.org/data).

#### Process

For each donor, one cell line was selected for the genetics-first prediction according to the following criteria: use primary tissue data if available, otherwise use the iPSC cell line with the minimum changes from the origin cell as measured by number of differences per Mbp, and excluding those where the pluritest or custom CNV check is missing. The result was that 437 cell lines from different donors were selected and the corresponding exome files processed using the 1KGP3 genomes as background, predicting from a set of 5,805 possible GO terms.

#### Evaluation

There was no confirmation step as with DTC and DDD, so no phenotype data was used. A list of phenotypes with several predicted outliers was examined to find one potentially verifiable by experiment. The list was initially narrowed to four candidates that could be tested on iPS-derived macrophage cells and two candidates that could be tested directly in iPS cells. Expression analysis of genes harboring the variants of interest uncovered a lack of expression for GO:0002741 and GO:1900025 in macrophage, so these were eliminated. The variants implicated in terms GO:0035718 and GO:0002840 are involved in cell signalling (e.g. from the thyroid) and were eliminated as too experimentally complicated. One term, GO:1901223, was excluded due to the lack of availability of a differentiated cell line for a key donor with the variant. Finally, GO:0010826 was selected for experimental validation because five iPS cell lines predicted as outliers were available.

#### Experimental test for GO:0010826

The Hoik-1 (HPSI0314i-hoik_1), Sehp-2 (HPSI0115i-sehp_2) and Kegd-2 (HPSI0614i-kegd_2) cell lines were selected as control. The Suul-1 (HPSI0514i-suul_1), Yoch-6 (HPSI0215i-yoch_6), Boqx-2 (HPSI0115i-boqx_2), Zapk-3 (HPSI0114i-zapk_3) and Iuoc-2 (HPSI0516i-iuoc_2) cell lines were tested. γ-tubulin was used as a centriole marker^45^. 2.5 × 10^4^ cells were plated onto coverslips maintained in 24-well multiwell plates and grown for 2 days. Cells were fixed in cold methanol for 5 min, rinsed, and incubated with 3% (wt/vol) BSA for 1 h. Cells were incubated with γ-tubulin antibody (Sigma-Aldrich, T5326), washed and incubated with the appropriate fluorescent secondary antibody conjugated to Alexa 555 (Invitrogen). DAPI (Thermo Fischer Scientific) was used as counterstain. Cells were mounted in coverslips using ProLong Gold antifade reagent (Thermo Fisher Scientific). Images were acquired with a Nikon A1R confocal microscope. Brightness and contrast were optimised with ImageJ (National Institutes of Health) and Photoshop (Adobe Systems). Quantifications of centrioles were performed manually using ImageJ. Dilutions: the γ-tubulin antibody was used at 1:5000, the secondary antibody at 1:500 and DAPI was used at 1:2000.

### Association and confounders

#### Confounding variables

Twelve potentially confounding variables were analysed on the DTC cohort data: sex, the first 10 principal components of ancestry, and the genotype array type. Each of the variables was assessed as a predictor on the DTC cohort under the same permutation test as our outlier scores. The correlation coefficients and t-test statistics were also calculated between each variable and the outlier scores for every phenotype. Whilst there is no theoretical reason to believe that predictions from a non-associative method should be confounded by covariates of association, we nevertheless checked this against the above statistics. PERL and packages PDL and Statistics were used as well as R package glm.

#### Variant associations

There is no expectation that methods based on association will perform well under the same conditions, e.g. high false discovery rate, as the predictor exemplified in this work. However, as a reference point we calculated GWAS statistics on the DTC cohort data using PLINK^46^ and subjected them to a 1000 iteration permutation test as in Fig. 3d. Python packages SciPy and NumPy were used here and in other parts of the work. Each phenotype was treated as a cohort with the answers to questions determining case vs control classification. The confounding variables above were used as covariates for the analysis. Within the framework of our reverse approach, predictor scores were used to allocate some of the questions, so to simulate whether the selection of questions contributes to the positive GWAS results, we also repeated the analysis with data points scoring > 0.022 removed. The GWAS results are summarised in Supplementary Fig. 2. GWAS were performed using a standard approach, as outlined below.

(i) DTC genomes quality check. We started with autosomal, bi-allelic SNPs in the DTC participants that had missingness <1% and those passing an AB ratio binomial test with *Z*-score <3 for the 1000 genomes project phase 3 data (1KGP3) overall and EUR superpopulation (from GenomePrep). (ii) High-quality common SNPs selection. We restricted variants to having frequency >5% in the 1KGP3, and excluded variants in complex regions from https://genome.sph.umich.edu/wiki/Regions_of_high_linkage_disequilibrium_(LD) and variants where the ref/alt combinations was CG or AT. We removed all SNPs which were out of Hardy Weinberg Equilibrium (HWE) with a *p*-value cut-off of pHWE < 1e-8. We LD-pruned using PLINK2 with *r2* = 0.1 and 500kb windows. The resulting 22,103 aggregate high-quality sites were used for kinship and genetic components analysis (PCA). (iii) Kinship and ancestry calculation. Data was merged with 1KGP3 and kinship coefficients were calculated among all pairs of samples using PLINK2 and its implementation of the KING robust algorithm. A kinship cutoff of 0.0884 was used to select unrelated individuals (-king-cutoff). Ancestry was inferred via PCA on unrelated 1KGP3 individuals with GCTAv1.93 (plink2) using HQ common SNPs. QC of variants included plink v1.9: HWE deviations exclude *p*-value <(1e-20), multi-allelic variants were excluded and we filtered variants by missingness <0.02. (iv) GWAS. A total of 4946035 associations were calculated and the multi-phenotype association significance level after multiple hypothesis correction is 1.01 x 10-8. GWAS was run for each phenotype term using ‘Yes’ answers as cases ‘No’ answers as controls. Covariate analysis was implemented with logistic regression of PLINK1.9; sex was imputed (female 1132, male 848, ambiguous 73), and the first 10 PCs from the genetic ancestry analysis were used as well as the version of the genotyping array were used. The permutation plots were generated by running the association on 1000 randomisations of the data and plotted as per Fig. 3d.

### Reporting summary

Further information on research design is available in the Nature Portfolio Reporting Summary linked to this article.

## Supplementary information

Supplementary Information

Description of Additional Supplementary Files

Supplementary Data 1-5

Reporting Summary

## Data Availability

All data supporting the findings described in the manuscript are available in the article, supplementary information files or from the corresponding author on request. Additionally, supplementary data are made available in an interactive, searchable format via the project webpage at https://supfam.org/nomaly. The DTC cohort data may not be made publicly available because participants are not consented for this, but on application to the corresponding author, requests falling within the constraints of ethical approval granted for the project will be responded to within 14 days. DTC data can be made available under MTA to academic organisations subject to MRC institutional approval and compliance with all relevant data protection laws and requirements. Access is time-limited because we are required to delete all participant data when our work on the DTC cohort ends, which could be before the end of 2023. The database of questions corresponding to 5,857 ontology terms (Supplementary Data 3) is also available via the resources webpage (above) and may be a valuable resource for other studies. The similarity mapping, by information content, of all HPO terms that are close to the HPO terms used in clinical annotations by DDD (Supplementary Data 4) are also made available on the resources webpage. All data on the resources webpage are also available for download in JSON format. Access to 1KGP3, DDD and HipSci cohorts is only available via those projects directly. Specifically: access to 1KGP3 is made publicly available by the International Genome Sample Resource (IGSR), with data sets accessible from the data portal (https://www.internationalgenome.org/data-portal). The DDD cohort data is available from the European Genome-phenome Archive (EGA, https://ega-archive.org/), with the study ID EGAS00001000775. To access these data sets, please contact datasharing@sanger.ac.uk. An overview of HipSci cell lines and assay data that are publicly available is available on the cell lines and data browser (https://www.hipsci.org/data). It also provides links to publicly available HipSci data in the EBI data archives. To access the managed-access genetic and genomic data in HipSci, please follow the steps stated in the data browser (https://www.hipsci.org/data#overview), and apply via the Wellcome Trust Sanger Institute’s Electronic Data Access Mechanism (https://www.sanger.ac.uk/legal/DAA).

## Code availability

Code for the preparation, parsing and classification of DTC data files is available via the GenomePrep^38^ resource already published as an ancillary output of this work that could be of value to others. The website running the pipeline providing questions to participants for the DTC study is available as a free service for up to 500 genotype files per submission (~24hr turnaround), and on request to run on larger datasets; we wish to collaborate with as many genetic cohorts as possible to provide predictions that can be made available within each of their data sharing portals to their registered users. The code used for the phenotype prediction to exemplify the Nomaly framework is not available for download. Individual applications for a license for non-commercial use are possible via the resources webpage (https://supfam.org/nomaly) to be considered on a case-by-case basis per project. Pipeline scripts behind the DTC cohort website are available via the resources website on request (not available for direct download due to web security risks). Scripts for conducting the permutation tests, generating the statistics for covariates and producing Fig. 5 are available for direct download via the resources website.
