# Supplementary material for: Hypothesis-free phenotype prediction within a genetics-first framework
Source: Nat Commun. 2023 Feb 17;14:919. doi: 10.1038/s41467-023-36634-6 (PMC9938118; doi:10.1038/s41467-023-36634-6)
Supplement: Supplementary file 1 — Supplementary Information [file 41467_2023_36634_MOESM1_ESM.pdf]

Supplementary Information

Hypothesis-free phenotype prediction within a genetics-first framework

Chang Lu, Jan Zaucha, Rihab Gam, Hai Fang, Ben Smithers, Matt E. Oates, Miguel Bernabe-Rubio, James Williams, Natalie Thurlby, Arun Prasad Pandurangan, Himani Tandon, Hashem Shihab, Kalaivani Raju, Minkyung Sung, Adam Sardar, Bastian Greshake Tzovoras, Davide Danovi and Julian Gough*

Supplementary Figures.

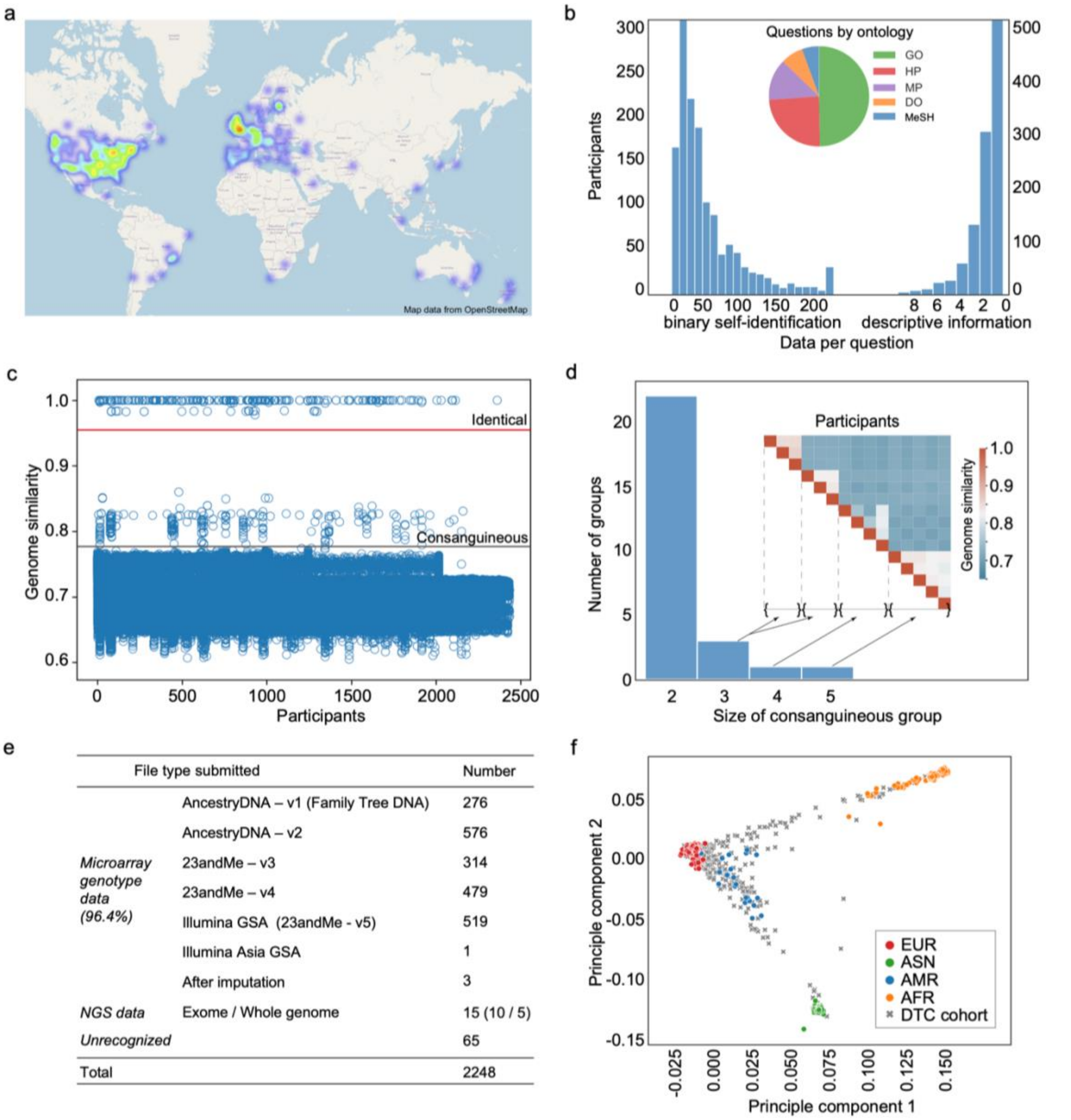

File type submitted		Number
	AncestryDNA – v1 (Family Tree DNA)	276
	AncestryDNA – v2	576
Microarray genotype data (96.4%)	23andMe – v3	314
	23andMe – v4	479
	Illumina GSA (23andMe - v5)	519
	Illumina Asia GSA	1
	After imputation	3
NGS data	Exome / Whole genome	15 (10 / 5)
Unrecognized		65
Total		2248

Supplementary Figure 1 - Overview of the direct-to-consumer (DTC) cohort. (a) The geo-spatial distribution of 2,248 participants. (b) Summary of the data collected for questions with at least one positive answer, including the number of binary answers to questions for self-identifying phenotype (left), number of descriptive free-text comments left per participant (right), and the breakdown of questions by ontology (pie chart) between: GO, gene ontology[1,2]; HP, human phenotype ontology[3]; MP, mammalian phenotype[4]; DO, disease ontology[5]; MeSH, mesh subject headings ontology[6]. (c) Pairwise similarities between all DNA data files. Files are considered to be genetically identical if over 97% of variants are the same, and consanguineous if above 78%. (d) Consanguineous groups in the data. (e) The types and quantity of files submitted by participants, identified using GenomePrep[7]. (f) The ethnicity of participants mapped to the 1000 Genomes project[8] ethnicity group principal components, labeled: EUR, European; ASN, East Asian; AMR, mixed American; AFR, African. Panel (a) was created with ipinfo (https://github.com/ipinfo/python) and the map came from OpenStreetMap (https://www.openstreetmap.org/), which is open data, licensed under the Open Data Commons Open Database License (ODbL) by the OpenStreetMap Foundation (OSMF)."

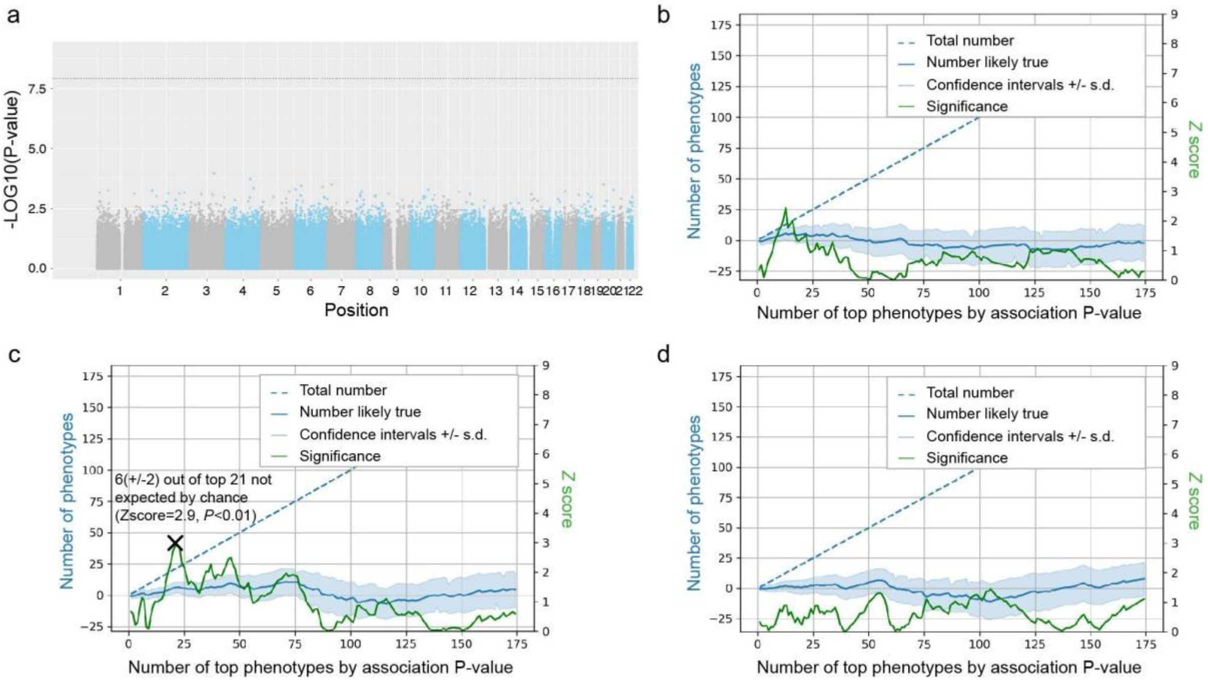

Supplementary Figure 2 - Association scores on the DTC cohort. (a) Manhattan plot of aggregate associations, corrected for covariates. Allele-based association statistics on 351 phenotypes were merged and shown by genome position, and multiple-hypothesis corrected P-value = 1.01E-8 (dashed line). (b-d) Performance evaluation using a similar procedure as in Figure 3d. Z scores (significance) were derived from testing the null hypothesis that similar results can be obtained if scores are assigned randomly. (b) Performance of uncorrected association scores on the permutation test. (c) Improved association performance on the permutation test after correcting for covariates. (d) Decreased performance on the permutation test simulating the removal of performance added by the selective questioning derived from predictor scores.

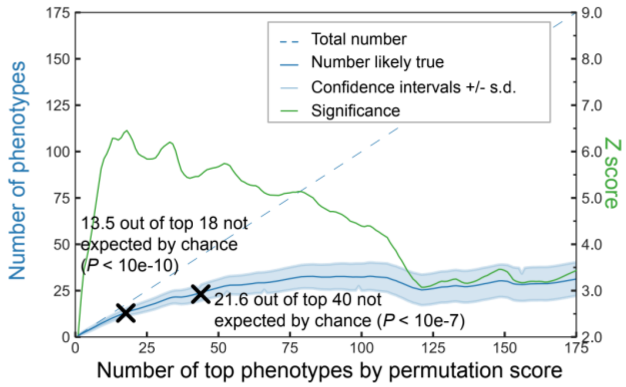

Supplementary Figure 3 - Performance on imputed data from the DTC cohort. Performance evaluation using a similar procedure as in Figure 3d, with the difference being that the results were calculated on data imputed from the original data used in Figure 3d. At the same point shown in Figure 3d, 21.5 out of the top 40 phenotypes are likely to be true, but the greatest significance is found in the top 18 where 13.5 are likely to be true. The performance is very similar to that without imputation, and there are only 4 terms in the top 40 that change.

Supplementary References

1. Ashburner, M. *et al.* Gene ontology: Tool for the unification of biology. *Nature Genetics* vol. 25 25–29 Preprint at https://doi.org/10.1038/75556 (2000).
2. Carbon, S. *et al.* The Gene Ontology resource: Enriching a GOld mine. *Nucleic Acids Res* **49**, D325–D334 (2021).
3. Köhler, S. *et al.* The human phenotype ontology in 2021. *Nucleic Acids Res* **49**, D1207–D1217 (2021).
4. Smith, C. L. & Eppig, J. T. The mammalian phenotype ontology: Enabling robust annotation and comparative analysis. *Wiley Interdiscip Rev Syst Biol Med* **1**, 390–399 (2009).
5. Schriml, L. M. *et al.* Disease Ontology: a backbone for disease semantic integration. *Nucleic Acids Res* **40**, D940–D946 (2012).
6. Lipscomb, C. E. Medical Subject Headings (MeSH). *Bull Med Libr Assoc* **88**, 265 (2000).
7. Lu, C., Greshake Tzovaras, B. & Gough, J. A survey of direct-to-consumer genotype data, and quality control tool (GenomePrep) for research. *Comput Struct Biotechnol J* **19**, 3747–3754 (2021).
8. Sudmant, P. H. *et al.* An integrated map of structural variation in 2,504 human genomes. *Nature* **526**, 75–81 (2015).
