# Supplementary material for: Hypothesis-free phenotype prediction within a genetics-first framework
Source: Nat Commun. 2023 Feb 17;14:919. doi: 10.1038/s41467-023-36634-6 (PMC9938118; doi:10.1038/s41467-023-36634-6)
Supplement: Supplementary file 2 — Description of Additional Supplementary Files [file 41467_2023_36634_MOESM2_ESM.pdf]

Additional Supplementary Files are provided, but also via searchable interactive tables online which include Json format downloads (https://supfam.org/nomaly/).

File Name:
Supplementary Data 1
Description:
List of the top-performing ontology terms in DTC cohort. (a) For each ontology term, showing the corresponding question, answer statistics, types of confirmed predictions, and contributing variants for predictions. The high-scoring variants were evaluated using combined information of rarity, zygosity and deleteriousness. Overall yes-rate below 5%. (b) Similar to a, control yes-rate below 5% (overall yes-rate above 5%).

File Name:
Supplementary Data 2
Description:
List of matched phenotype predictions in DDD cohort. For each person with at least one clinically annotated HPO terms positively predicted, showing the matched annotation, the predictions that exactly/closely matched, types of matched predictions, and contributing variants. Diagnostic status: 0 if no DNM nor diagnosis is found for the person, 1) if DNM is found but not interpreted with DDG2P, 2) if a genetic diagnosis has been provided with DDG2P.

File Name:
Supplementary Data 3
Description:
Questions mapping. Questions were specifically designed, such that people who are outliers of the phenotype described by corresponding ontology term tend to answer Yes. Ontology terms were selected from several ontology databases, including the disease ontology (DO), medical subject headings (MeSH), human phenotype ontology (HPO), mammalian phenotype (MP) and gene ontology (GO) databases.

File Name:
Supplementary Data 4
Description:
Mapping of HPO terms to closely matched terms used in DDD annotations. For each HPO term, the list of exactly or closely matched term(s) that are used by the 1133 trio DDD phenotype annotations is shown. Blank cells mean that for this HPO term, no corresponding closely-matched term was used in any DDD clinical annotation.

File Name:
Supplementary Data 5
Description:
List of the top-performing ontology terms by association in DTC cohort. For each ontology term, showing the corresponding question, answer statistics, and the variant.
