# Supplementary material for: Hypothesis-free phenotype prediction within a genetics-first framework
Source: Nat Commun. 2023 Feb 17;14:919. doi: 10.1038/s41467-023-36634-6 (PMC9938118; doi:10.1038/s41467-023-36634-6)
Supplement: Supplementary file 3 — Supplementary Data 1-5 [file 41467_2023_36634_MOESM3_ESM.zip › Supplementary Data/Supplementary Data 5.pdf]

Supplementary Table 5. List of the top-performing ontology terms by association analysis in DTC cohort. For each ontology term, showing the corresponding question, answer statistics, and the variant.

Ontology term	Question	Num answers: Yes(cases)/No(Controls)	Chr:pos_alt(GRCh37), rsid(link to dbSNP)
GO:0046548 retinal rod cell development	Have you or anyone in your family ever undergone electroretinography (ERG) showing retinitis pigmentosa (vision disorder) which is characterized by decreased night vision (nyctalopia), loss of the mid-peripheral visual field?	13/267	3:135351730_G, rs3850168
GO:0006465 signal peptide processing	Have you or anyone in your family been diagnosed with familial isolated hypoparathyroidism (rare genetic disorders) which is characterized by abnormal calcium metabolism due to deficient secretion of parathormone?	9/178	7:24480963_A, rs7799805
GO:1901223 negative regulation of NIK/NF-kappaB signaling	Anhidrotic/hypohidrotic ectodermal dysplasia - disorders result in the abnormal development of structures including the skin, hair, nails, teeth, and sweat glands. Has any child in your family ever been diagnosed with Anhidrotic/Hypohidrotic Ectodermal Dysplasia characterized by reduced ability to sweat, missing teeth and fine sparse hair?	11/292	4:148891027_T, rs17024235
HP:0001265 Hyporeflexia	Have you been diagnosed with hyporeflexia (reduction in reflexes) such as the knee-jerk reaction?	8/246	17:15554561_A, rs2074890
HP:0003271 Visceromegaly	Have you ever had an computed tomography (CT) scan showing abnormally increased size of the organs such as liver, spleen, stomach, kidneys, or pancreas in the abdomen region or Have you been diagnosed with visceromegaly?	8/182	5:150930186_T, rs2278370
HP:0002123 Generalized myoclonic seizures	Do you suffer from generalized myoclonic seizures which are brief shock-like jerks of a muscle or group of muscles?	8/165	1:199150453_T, rs6684332
HP:0007210 Lower limb amyotrophy	Muscular atrophy affecting the lower limb. Do you have muscle pain in lower limb or Have you ever had Nerve conduction velocity(NCV) or Electromyography(EMG) analysis resulted in lower limb amyotrophy?	10/241	15:25841802_G, rs3850427
GO:0060837 blood vessel endothelial cell differentiation	Ischemic wounds occur as a direct result of blocked blood flow to medium and small vascular beds in the body. Have you noticed ischemic chronic wounds on legs, feet and toes?	8/165	4:48036711_G, rs10517204
GO:0035937 estrogen secretion	Have you ever had blood test showing increased level of estrogen (Hyperestrogenism) or breast enlargement in boys or men (gynecomastia) due to estrogen testosterone imbalance ?	6/202	8:19213714_A, rs17128272
GO:0071236 cellular response to antibiotic	Antibiotic drugs are penicillin, cephalosporin, erythromycin Are you taking antibiotic medication frequently or Do you experience decreased response to any antibiotic medication like antibiotic resistance?	8/162	8:124591674_G, rs16898594
GO:0051560 mitochondrial calcium ion homeostasis	Have you ever been diagnosed with amyotrophic lateral sclerosis (ALS) (motor neurone disease) through electromyography (EMG) or been suffering from symptoms like stiff muscles, muscle twitching, and muscle weakness?	8/171	12:8023500_A, rs12372273
GO:1905153 regulation of membrane invagination	Chronic granulomatous disease - is an inherited primary immunodeficiency disease (PIDD) which increases the body's susceptibility to infections caused by certain bacteria and fungi. Have you ever had any blood test showing elevated levels of white blood cells (WBC) and diagnosed with Chronic granulomatous disease, or do you suffer from symptoms like recurrent infection, Persistent diarrhea and Chronic runny nose?	6/176	5:6550709_T, rs11960029
GO:0006303 double-strand break repair via nonhomologous end joining	Are you working in Nuclear power plant or nuclear fuel reprocessing plant or Have you been diagnosed with any type of cancer or Do you have symptoms like skin redness, hair loss, radiation burns, or acute radiation syndrome (acute illness caused by irradiation) when exposure to ionizing radiation (like X-ray, ionising radiation)?	6/118	8:60024449_A, rs392450
MP:0001731 abnormal postnatal growth	Incase of being a parent, Is your child have any abnormal growth characteristics?	6/171	12:30942174_A, rs2434118

Ontology term	Question	Num answers: Yes(cases)/No(Controls)	Chr:pos_alt(GRCh37), rsid(link to dbSNP)
GO:2000370 positive regulation of clathrin-mediated endocytosis	Have you or anyone in your family diagnosed with muscle biopsy which may indicate centronuclear myopathy manifested by decreased muscle tone, muscle weakness, speech difficulties, muscle pain, leg cramps, difficulty walking?	9/273	21:16520832_A, rs2823093
GO:0003170 heart valve development	Bicuspid aortic valve (BAV): Two of the leaflets of the aortic valve fuse during development in the womb resulting in a two-leaflet valve (bicuspid valve) instead of the normal three-leaflet valve (tricuspid) Aortic valve calcification is a condition in which calcium deposits form on the aortic valve in the heart. Have you ever had Cardiac Magnetic Resonance Imaging (MRI) or Electron-beam computed tomography (EBCT) analysis showing defects in heart valve or Have you or anyone in your family been diagnosed with any heart valve diseases like bicuspid aortic valve, aortic valve calcification?	5/106	5:36422893_C, rs10512652
HP:0000326 Abnormality of the maxilla	Have you undergone dental X-ray analysis showing any abnormalities in the upper jaw bone or maxilla like prognathism (bulging out of lower jaw), retrognathia (abnormal posterior positioning of jaws)?	6/114	12:1753225_T, rs2240510
GO:0033138 positive regulation of peptidyl-serine phosphorylation	Do you suffer from impaired cognition, sleep disorders, visual hallucinations which are the symptoms of a-Synucleinopathy (neurodegenerative disease)?	5/118	2:192381934_C, rs2356656
GO:0010823 negative regulation of mitochondrion organization	Have you or anyone in your family ever been diagnosed with Leber hereditary optic neuropathy(eye disorder) which is characterized by cardiac conduction abnormalities, muscles contract uncontrollably (dystonia), blurring or clouding of vision in one eye?	5/190	6:34841330_G, rs3800453
HP:0000069 Abnormality of the ureter	Have you ever had ultrasonography analysis showing any ureter abnormalities like uterine cancer?	6/193	5:156589585_G, rs31208
MP:0002412 increased susceptibility to bacterial infection	Do you suffer from recurrent bacterial infection like cholera, pneumonia?	6/120	16:81010946_C, rs16954368
