# Supplementary material for: Hypothesis-free phenotype prediction within a genetics-first framework
Source: Nat Commun. 2023 Feb 17;14:919. doi: 10.1038/s41467-023-36634-6 (PMC9938118; doi:10.1038/s41467-023-36634-6)
Supplement: Supplementary file 3 — Supplementary Data 1-5 [file 41467_2023_36634_MOESM3_ESM.zip › Supplementary Data/Supplementary Data 1.pdf]

Supplimentary Table 1. List of the top-performing ontology terms in DTC cohort.

1a. For each ontology term, showing the corresponding question, answer statistics, types of confirmed predictions, and contributing variants for predictions. The high-scoring variants were evaluated using combined information of rarity, zygosity and deleteriousness. Only ontology terms whose percentage of positive answers is below 5% are included.

Ontology term	Question	Num answers: Yes/Total (Predicted yes)	Types predicted	Superfamily/Pfam Domain	Gene, chr:pos_ref/alt(GRCh37)-Genotype (MAF in 1000G) *high-scoring variant
DO:DOID:896 metal metabolism disorder	Metal metabolism disorder is an inherited metabolic disorder that involves metabolic disturbances in the processing or distribution of dietary minerals. Anyone in your family been diagnosed with metal metabolism disorder?	1/46 (1)	1-b	Immunoglobulin	*HFE, 6:26093141_G/A-AA (1.26%)
				Voltage-gated potassium channels	CACNA1S, 1:201052310_A/T-AT (12.72%)<br>TPCN2, 11:68846399_A/T-AT (9.96%)
MP:0000286 abnormal mitral valve morphology	Abnormal Mitral valve regurgitation is a backflow of blood caused by failure of the heart's mitral valve to close tightly. Have you been diagnosed with abnormal Mitral valve regurgitation through Echocardiography (ECG) or Electrocardiography (EKG) test?	2/50 (2)	1-b<br>1-a	Metalloproteases ("zincins") catalytic domain	*ADAMTS13, 9:136293770_G/C-GC (0.0%)<br>ADAM7, 8:24339679_G/A-AA (26.14%)<br>*ADAM7, 8:24339679_G/A-AA (26.14%)
MP:0010402 ventricular septal defect	Ventricular septum is the wall dividing the lower chambers of the heart. Have you undergone Echocardiography showing any defect in ventricular septum?	1/47 (1)	1-a	SMAD MH1 domain	*SMAD9, 13:37453762_A/G-AG (0.1%)
GO:1901213 regulation of transcription from RNA polymerase II promoter involved in heart development	Have you ever had magnetic resonance imaging (MRI) showing thickened heart muscle which may indicate cardiac hypertrophy (abnormal heart muscle enlargement) which is characterized by shortness of breath, general fatigue, fainting, palpitations?	2/85 (1)	1-b	Cystine-knot cytokines	*GDF3, 12:7842773_G/A-AG (0.04%)<br>*LHB, 19:49519905_A/G-GG (5.79%)
GO:0014854 response to inactivity	Do you have a medical history of polio attack or have you ever undergone electromyography (EMG) showing signs of nerve damage orneuromuscular examination showing signs of residual weakness and atrophy of muscles?	3/80 (2)	1-a<br>1-a	F-box domain	*FBXW12, 3:48414274_C/T-TT (0.6%)<br>*FBXW12, 3:48414274_C/T-CT (0.6%)

Ontology term	Question	Num answers: Yes/Total (Predicted yes)	Types predicted	Superfamily/Pfam Domain	Gene, chr:pos_ref/alt(GRCh37)-Genotype (MAF in 1000G)<br>*high-scoring variant
GO:0031646 positive regulation of neurological system process	Have you or anyone in your family ever undergone blood test showing very high levels of immunoglobulin E (IgE) which may indicate job's syndrome (immune disorder), characterized by recurrent cold, unusual skin rashes, severe lung infections?	2/66<br>(2)	2-b<br>2-b	EGF/Laminin	*MASP2, 1:11105542_C/T-CT (0.32%)<br>*LTBP1, 2:33567971_C/T-CT (0.4%)<br>LAMA5, 20:60910124_T/G-GT (1.14%)<br>EGF, 4:110914427_A/T-AT (7.95%)<br>HEG1, 3:124728626_A/G-AA (37.18%)<br>LAMA5, 20:60899196_C/T-CT (1.02%)<br>*LAMC3, 9:133936571_C/G-GG (0.0%)<br>USH2A, 1:216462662_T/A-AT (3.45%)<br>LAMA4, 6:112522852_G/A-AG (3.09%)<br>SELL, 1:169676486_A/G-AG (13.3%)
				EGF/Laminin<br>Growth factor receptor domain	CRTAC1, 10:99640120_C/T-CT (0.7%)
MP:0009445 osteomalacia	Osteomalacia is the softening of the bones caused by impaired bone metabolism primarily due to inadequate levels of available phosphate, calcium, and vitamin D, or because of resorption of calcium. Have you undergone X-ray analysis showing the presence of osteomalacia?	2/96<br>(2)	1-b<br>2-b	EGF/Laminin	*MASP2, 1:11105542_C/T-CT (0.32%)<br>SELL, 1:169676486_A/G-GG (13.3%)
				Metalloproteases ("zincins") catalytic domain	ADAM7, 8:24339679_G/A-AA (26.14%)<br>ADAM7, 8:24339679_G/A-AG (26.14%)
				EGF/Laminin<br>Growth factor receptor domain	CRTAC1, 10:99640120_C/T-CT (0.7%)
HP:0000280 Coarse facial features	Does your face seems to be coarse like to having large, bulging head, large lips and tongue and small, widely spaced malformed teeth etc?	1/83<br>(1)	2-b	Alkaline phosphatase-like	*ARSH, X:2945477_C/T-TT (3.79%)<br>ARSF, X:3030403_C/T-TT (5.38%)<br>ARSG, 17:66364804_T/C-CC (35.94%)
				ARM repeat	AP4B1, 1:114438951_A/G-GG (37.7%)
HP:0002605 Hepatic necrosis	Does your blood test shows that you have a elevated levels of liver enzymes which is the main symptom of hepatic necrosis or Have you been diagnosed with hepatic necrosis?	2/48<br>(2)	2-b<br>2-b	Thioredoxin-like	*PDIA2, 16:335373_C/T-TT (8.11%)<br>GSTZ1, 14:77794283_T/C-TC (14.7%)<br>PDIA2, 16:334890_G/A-AA (10.2%)<br>*PDIA2, 16:335373_C/T-TC (8.11%)<br>GSTZ1, 14:77793237_G/A-AG (3.06%)<br>GSTZ1, 14:77793207_G/A-AA (31.15%)
				FAD/NAD(P)-binding domain	*FMO2, 1:171168545_T/C-CC (11.66%)<br>PCYOX1, 2:70488470_C/T-TC (5.21%)<br>FMO2, 1:171154959_A/G-AG (9.21%)<br>FMO2, 1:171174531_A/G-AG (15.97%)
GO:0048708 astrocyte differentiation	Has any child in your family ever been diagnosed with Alexander disease (disorder of the nervous system) which is characterized by enlarged brain and head size (megalencephaly), seizures, stiffness in arms/ legs (spasticity), intellectual disability, developmental delay?	1/60<br>(1)	2-b	Metallothionein	*MT4, 16:56601722_T/C-TT (13.4%)<br>*MT4, 16:56601720_A/G-AA (14.16%)
				ARM repeat	*ARMC10, 7:102715804_C/T-TT (7.35%)<br>*CUL7, 6:43017728_C/A-AC (0.34%)<br>WDFY4, 10:49933974_T/C-CT (14.02%)<br>HEATR5A, 14:31819047_C/T-CT (19.49%)
				p53-like transcription factors	*NFATC4, 14:24842563_A/C-AC (0.08%)

Ontology term	Question	Num answers: Yes/Total (Predicted yes)	Types predicted	Superfamily/Pfam Domain	Gene, chr:pos_ref/alt(GRCh37)-Genotype (MAF in 1000G) *high-scoring variant
DO:DOID:11030 corneal edema	Corneal edema is the swelling of the cornea following ocular surgery, trauma, infection, inflammation as well as a secondary result of various ocular diseases. Have you been diagnosed with corneal edema?	3/60 (3)	1-a<br>1-a<br>1-a	Cystine-knot cytokines	*GDF3, 12:7842773_G/A-AG (0.04%)<br>*GDF9, 5:132197286_G/A-AG (0.28%)
GO:2000516 positive regulation of CD4-positive, alpha-beta T cell activation	Have you ever had blood test showing low level of albumin which may indicate hypoalbuminemia or Do you suffer from edema (buildup of fluid) in your legs or face, skin that's rougher or drier than normal, not having much of an appetite ?	2/49 (1)	2-b	Annexin	*ANXA9, 1:150958836_A/G-GG (6.35%)<br>ANXA11, 10:81926702_G/A-AA (39.22%)<br>ANXA10, 4:169083694_A/C-AA (46.69%)
				DEATH domain	CARD6, 5:40841741_C/T-CT (4.81%)
				Protein kinase-like (PK-like)	VRK2, 2:58316814_A/G-GG (30.19%)<br>GRK5, 10:121196335_G/A-AG (12.58%)
				Cysteine-rich domain	CHN2, 7:29519929_A/G-AG (12.72%)
GO:0016447 somatic recombination of immunoglobulin gene segments	Have you or anyone in your family ever had blood test showing decreased levels of immunoglobulins which may indicate immunodeficiency–centromeric instability–facial anomalies syndrome (ICF syndrome) (rare immune disorder), characterized by increased distance between bodily parts (hypertelorism), skin fold of the upper eyelid, unusually large tongue?	1/60 (1)	2-b	MutS domain II	*MSH6, 2:48027194_T/C-CC (0.0%)
				DNA repair protein MutS domain II	*MSH2, 2:47639670_A/G-GG (0.0%)
				DNA repair protein MutS domain III	*MSH2, 2:47690259_G/C-CC (0.0%)<br>*MSH2, 2:47693873_A/C-CC (0.0%)<br>MSH6, 2:48027778_A/G-GG (0.0%)
				P-loop containing nucleoside triphosphate hydrolases	*ABCC12, 16:48122456_C/T-CT (1.54%)<br>ABCC12, 16:48121912_A/G-AG (4.77%)<br>ABCC11, 16:48201432_T/C-TC (17.01%)
				DNA repair protein MutS domain I	*MSH6, 2:48026560_G/A-AA (0.0%)
				BRCT domain	BRCA1, 17:41219651_T/C-CC (0.0%)
				RING/U-box	TRIM40, 6:30104989_C/A-CA (0.92%)
MESH:D010182 Pancreatic Diseases	Have you been diagnosed with any pancreatic diseases like pancreatitis (pancreas inflammation), pancreatic cyst, cystic fibrosis etc?	2/77 (1)	2-b	EGF/Laminin	*FBN3, 19:8168538_C/T-CT (0.08%)<br>EGF, 4:110914427_A/T-AT (7.95%)
				Growth factor receptor domain	*NOTCH4, 6:32180623_C/A-AC (1.48%)
				HLH helix-loop-helix DNA-binding domain	*MYOG, 1:203054651_G/A-AG (1.2%)
GO:2000104 negative regulation of DNA-dependent DNA replication	Have you ever been diagnosed with magnetic resonance imaging (MRI), shows defects in eye movements which may indicate ophthalmoplegia manifested by stroke, infection, abnormal eye movements, muscle weakness?	2/113 (1)	1-a	F-box domain	*FBXW12, 3:48414274_C/T-TT (0.6%)

Ontology term	Question	Num answers: Yes/Total (Predicted yes)	Types predicted	Superfamily/Pfam Domain	Gene, chr:pos_ref/alt(GRCh37)-Genotype (MAF in 1000G) *high-scoring variant
GO:0036314 response to sterol	Have you ever underwent electrocardiogram showing irregular electrical activity of heart and been diagnosed with heart attack which is associated with symptoms like tightness or pain in the chest, neck, back or arms, lightheadedness, abnormal heartbeat, anxiety, shortness of breath and shoulder discomfort?	1/33 (1)	2-b	Cytochrome P450	*CYP4B1, 1:47282772_C/T-TT (15.83%)<br>CYP2C9, 10:96702047_C/T-CT (4.79%)<br>CYP2C8, 10:96798749_T/C-CT (4.57%)<br>CYP4A22, 1:47609489_T/C-TT (41.93%)
				beta-beta-alpha zinc fingers	ZNF836, 19:52659872_T/C-CT (1.3%)<br>ZNF22, 10:45499009_A/G-AG (3.44%)<br>ZNF596, 8:196274_T/G-GG (16.93%)<br>ZNF695, 1:247151035_T/G-GT (8.63%)<br>ZNF30, 19:35435006_G/A-GG (34.76%)<br>ZNF10, 12:133732512_A/G-AG (6.73%)
				Integrin alpha N-terminal domain	ITGA1, 5:52201722_C/T-CT (9.61%)
				Cystine-knot cytokines	IL17F, 6:52101844_T/C-CT (3.31%)
GO:0000724 double-strand break repair via homologous recombination	Have you been diagnosed with photokeratitis (cornea inflammation) which is associated with symptoms like teary eyes, eye pain, swollen eyelids, feeling of sand in the eye, hazy and decreased vision ?	1/28 (1)	2-b	L domain-like	*LRR1, 14:50074520_C/T-TT (17.23%)
				FAD/NAD(P)-binding domain	*PCYOX1, 2:70488470_C/T-TT (5.21%)
				RNI-like	NOD2, 16:50756540_G/C-CG (0.46%)<br>NLRP13, 19:56419263_T/C-CT (11.98%)
				P-loop containing nucleoside triphosphate hydrolases	XRCC3, 14:104165753_G/A-AA (21.68%)<br>ENSG00000267618<br>RAD51D, 17:33433487_C/T-TT (9.5%)<br>XRCC2, 7:152346007_C/T-CT (5.25%)
MP:0009701 abnormal birth body size	Anyone in your family have any abnormal body size during birth?	2/73 (1)	1-a	Insulin-like	*INSL3, 19:17927755_G/A-AG (0.0%)
GO:0030510 regulation of BMP signaling pathway	Fibrodysplasia ossificans progressiva (FOP) - abnormal development of bone in areas of the body where bone is not normally present. Have you or anyone in your family ever had blood test showing increased level of alkaline phosphatase which may indicate fibrodysplasia ossificans progressiva or or Do you have symptoms like loss of mobility or difficulty in speaking and eating due to Inability to fully open the mouth, episodes of muscle swelling and inflammation ?	1/30 (1)	2-b	Alkaline phosphatase-like	*ARSD, X:2835863_G/T-TG (0.0%)<br>ARSG, 17:66364804_T/C-CC (35.94%)<br>SGSH, 17:78184679_C/T-TC (6.35%)
				L domain-like	*LRR1, 14:50074520_C/T-TT (17.23%)
				Cystine-knot cytokines	*LHB, 19:49519905_A/G-GG (5.79%)
				beta-beta-alpha zinc fingers	*ZNF439, 19:11979164_T/C-CC (10.02%)
				EGF/Laminin	*LAMC3, 9:133936571_C/G-GG (0.0%)
				SH3-domain	PLCG1, 20:39797465_T/C-TT (26.72%)
				von Willebrand factor type D domain	ZAN, 7:100374087_A/G-GG (24.52%)
				Concanavalin A-like lectins/glucanases<br>Growth factor receptor domain	SVEP1, 9:113209195_T/G-TG (11.34%)
				Concanavalin A-like lectins/glucanases	CLSTN2, 3:140178381_T/C-TC (3.02%)<br>FSD2, 15:83428192_C/T-TC (9.61%)
				Growth factor receptor domain	NOTCH4, 6:32185796_C/T-TC (3.12%)
				SAND domain-like	SP110, 2:231042276_A/G-GG (32.49%)
				Serine protease inhibitors	OTOGL, 12:80747242_A/G-AG (16.59%)

Ontology term	Question	Num answers: Yes/Total (Predicted yes)	Types predicted	Superfamily/Pfam Domain	Gene, chr:pos_ref/alt(GRCh37)-Genotype (MAF in 1000G) *high-scoring variant
HP:0000204 Cleft upper lip	Have you noticed any of your family members have openings or splits in the roof of the mouth and lip or Do anyone have cleft upper lip?	1/28 (1)	1-a	SMAD/FHA domain	*APLF, 2:68717350_A/T-AT (0.08%)
HP:0000277 Abnormality of the mandible	Have you ever undergone X-ray analysis showing any abnormalities in lower jaw bone like broad jaw?	1/36 (1)	2-b	Actin-like ATPase domain	*ACTB, 7:5569225_C/T-TT (0.0%)<br>*ACTR1B, 2:98275354_G/A-AG (2.38%)
				Tropomyosin	*HMMR, 5:162896650_C/T-TT (7.47%)
				WD40 repeat-like	*AHI1, 6:135751024_G/A-AG (1.14%)<br>*CFAP43, 10:105957714_A/G-GG (7.69%)<br>LRBA, 4:151199080_G/A-AG (14.22%)<br>CDC20B, 5:54410099_G/A-AG (16.95%)<br>WDR63, 1:85589842_A/G-AG (5.27%)<br>LLGL2, 17:73552185_G/A-AA (40.91%)
				Restriction endonuclease-like	ERCC4, 16:14041570_T/C-TC (0.0%)
				Growth factor receptor domain	FBN2, 5:127609633_G/A-AG (7.57%)
				EGF/Laminin	SELL, 1:169676486_A/G-AG (13.3%)
HP:0000269 Prominent occiput	Do you have prominent occiput or prominent posterior skull?	1/33 (1)	1-b	HLH helix-loop-helix DNA-binding domain	*MYOG, 1:203054651_G/A-GA (1.2%)<br>MESP2, 15:90320000_G/A-GA (7.85%)
GO:0008333 endosome to lysosome transport	Arthrogryposis-Renal dysfunction-Cholestasis (ARC) syndrome is a multisystem disorder, characterized by neurogenic arthrogryposis multiplex congenita, renal tubular dysfunction and neonatal cholestasis with low serum gamma-glutamyl transferase activity. Have you or anyone in your family been diagnosed with Arthrogryposis-Renal dysfunction-Cholestasis (ARC) syndrome?	1/24 (1)	1-b	SNARE-like	*AP1S3, 2:224642493_G/A-AG (0.24%)
				SNARE fusion complex	STX8, 17:9408381_C/T-CT (0.54%)
MP:0009672 abnormal birth weight	Incase of being a parent, Is your child have any abnormal body weight during birth?	2/57 (1)	1-a	Insulin-like	*INSL3, 19:17927755_G/A-AG (0.0%)

Ontology term	Question	Num answers: Yes/Total (Predicted yes)	Types predicted	Superfamily/Pfam Domain	Gene, chr:pos_ref/alt(GRCh37)-Genotype (MAF in 1000G) *high-scoring variant
MP:0001575 cyanosis	Have you noticed any bluish or purplish discoloration in your skin without any apparent cause or Have you been diagnosed with cyanosis?	4/86 (3)	2-b<br>2-b<br>2-b	EGF/Laminin	*FBN3, 19:8168538_C/T-CT (0.08%)<br>EGF, 4:110914427_A/T-AT (7.95%)<br>EGF, 4:110914427_A/T-TA (7.95%)<br>HEG1, 3:124728626_A/G-AA (37.18%)
				Growth factor receptor domain	*NOTCH4, 6:32180623_C/A-AC (1.48%)<br>*FBN1, 15:48764779_G/C-CC (0.0%)<br>NOTCH4, 6:32185796_C/T-TC (3.12%)<br>FBN2, 5:127609633_G/A-AG (7.57%)
				HLH helix-loop-helix DNA-binding domain	*MYOG, 1:203054651_G/A-AG (1.2%)
				Homeodomain-like	RAX2, 19:3771586_G/A-AG (0.6%)<br>CERS4, 19:8320555_G/A-AG (10.26%)
GO:0006032 chitin catabolic process	Have you ever been diagnosed with Cystic fibrosis (CF) (genetic disorder) through Sweat test and genetic testing or do you suffer from long-term issues include difficulty breathing and coughing up mucus?	1/24 (1)	2-b	(Trans)glycosidases	*GBA, 1:155208421_G/A-AA (0.0%)<br>GALC, 14:88442712_C/T-TC (7.95%)
				E set domains	*ARR3, X:69498482_C/G-GG (0.21%)<br>FLNC, 7:128488734_G/A-AG (6.25%)<br>KCNJ1, 11:128709126_A/G-AG (0.28%)<br>TGM6, 20:2375262_A/G-AG (12.2%)<br>KCNJ11, 11:17408630_C/T-CC (26.94%)
GO:0008592 regulation of Toll signaling pathway	Have you or anyone in family been diagnosed with familial cold autoinflammatory syndrome (periodic fever syndrome) which can cause recurrent, intermittent episodes of fever and rash that primarily follow exposure to cold?	1/111 (1)	2-b	Cysteine proteinases	*NAT1, 8:18079746_C/T-CT (0.12%)<br>NAT2, 8:18257854_T/C-CC (29.27%)<br>CAPN11, 6:44141088_G/A-AG (14.18%)<br>CAPN9, 1:230895340_C/A-AC (15.47%)<br>CTSB, 8:11710888_G/C-GG (39.6%)
				Trypsin-like serine proteases	*MST1, 3:49721532_G/A-AA (19.19%)<br>TPSG1, 16:1272038_G/A-AG (12.24%)<br>LPA, 6:160961137_T/C-CT (5.13%)<br>PRSS38, 1:228033197_A/G-AG (20.27%)<br>TMPRSS12, 12:51237816_G/A-AA (16.83%)<br>HGFAC, 4:3451109_G/A-AG (6.75%)
				Serpins	SERPINB13, 18:61264298_G/A-GG (13.98%)<br>SERPINB11, 18:61387333_T/A-AA (45.09%)<br>AGT, 1:230845794_A/G-AG (29.49%)<br>SERPINB11, 18:61387312_G/A-AA (45.11%)
HP:0000069 Abnormality of the ureter	Have you ever had ultrasonography analysis showing any ureter abnormalities like uterine cancer?	6/199 (1)	1-a	Glucocorticoid receptor-like (DNA-binding domain)	*ZYX, 7:143086010_C/T-CT (0.82%)
GO:0004792 thiosulfate sulfurtransferase activity	Have you ever had blood test or endoscopy analysis showing ulcerative colitis (colon ulcer) which may causes abdominal pain, diarrhea, weight loss, fever, anemia?	1/27 (1)	1-b	Activating enzymes of the ubiquitin-like proteins	*ATG7, 3:11400019_T/C-CC (3.0%)<br>*UBA7, 3:49845526_T/C-CT (2.76%)

Ontology term	Question	Num answers: Yes/Total (Predicted yes)	Types predicted	Superfamily/Pfam Domain	Gene, chr:pos_ref/alt(GRCh37)-Genotype (MAF in 1000G) *high-scoring variant
GO:0006021 inositol biosynthetic process	Have you ever been diagnosed with inositol deficiency which is characterized by symptoms like Vision and/or eye abnormalities, alopecia (patchy hair loss), fatty liver, loss of memory, constipation, high LDL cholesterol levels?	3/64 (2)	2-b<br>2-b	NAD(P)-binding Rossmann-fold domains	*G6PD, X:153763551_G/C-C (0.0%)<br>G6PD, X:153762655_T/A-A (0.0%)
				Glyceraldehyde-3-phosphate dehydrogenase-like C-terminal domain	*G6PD, X:153760649_C/G-G (0.0%)
				Carbohydrate phosphatase	INPP1, 2:191235610_A/G-AG (5.95%)
GO:0006284 base-excision repair	Have you ever had electromyography (EMG) or nerve conduction studies (NCS) or somatosensory evoked potential (SSEP) analysis resulted in nerve degeneration or Have you or anyone inyour family been diagnosed with Huntington's disease (progressive breakdown of nerve cells in the brain)?	2/47 (2)	2-b<br>1-b	Glucocorticoid receptor-like (DNA-binding domain)	*ZYX, 7:143086010_C/T-CT (0.82%)<br>FHL5, 6:97051593_G/A-AG (1.46%)
				S13-like H2TH domain	*NEIL2, 8:11643553_G/T-TT (5.29%)
				DNA/RNA polymerases	*RTL1, 14:101348572_C/T-CT (1.02%)
				Nucleotidyltransferase	POLL, 10:103340056_G/A-AG (9.98%)<br>*OAS3, 12:113379390_C/T-CT (0.68%)
				Nuclear receptor ligand-binding domain<br>Glucocorticoid receptor-like (DNA-binding domain)	HNF4A, 20:43042364_C/T-CT (2.4%)
				S-adenosyl-L-methionine-dependent methyltransferases	COMT, 22:19951271_G/A-AA (36.92%)
MP:0003920 abnormal heart right ventricle morphology	Have you ever had magnetic resonance imaging (MRI) scan that showing any morphological abnormality in heart right ventricle such as right ventricle degeneration, right ventricle aneurysm (weakening)?	2/65 (2)	2-b<br>1-b	Cystine-knot cytokines	*LHB, 19:49519905_A/G-GG (5.79%)
				Metalloproteases ("zincins") catalytic domain	ADAM7, 8:24339679_G/A-AA (26.14%)
HP:0000971 Abnormality of the sweat gland	Have you been diagnosed with any sweat gland disease like hyperhidrosis (excessive sweating), anhidrosis (inability to sweat normally)?	4/86 (1)	2-b	Connexin	*GJB3, 1:35250457_C/T-TT (1.3%)
				Prefoldin	KRT81, 12:52681925_A/C-CC (0.0%)
				Intermediate filament protein coiled coil region	KRT75, 12:52827608_C/T-CT (14.32%)<br>KRT76, 12:53167395_C/T-CT (16.39%)
				EGF/Laminin	LAMA5, 20:60897721_C/T-CT (14.96%)<br>UMODL1, 21:43510437_A/G-AG (0.0%)
				vWA-like	C2, 6:31903804_G/C-CG (2.97%)<br>VIT, 2:37035934_A/G-AG (18.03%)
				Intermediate filament protein	KRT15, 17:39674641_T/C-TT (26.72%)
				HMG-box	HMGB4, 1:34330067_A/C-AC (9.88%)

Ontology term	Question	Num answers: Yes/Total (Predicted yes)	Types predicted	Superfamily/Pfam Domain	Gene, chr:pos_ref/alt(GRCh37)-Genotype (MAF in 1000G) *high-scoring variant
HP:0002921 Abnormality of the cerebrospinal fluid	Cerebral Spinal Fluid (CSF) analysis requires CSF which is collected by lumbar puncture. It is usually performed by a doctor who is specially trained to collect CSF. Have you had Cerebral Spinal Fluid (CSF) analysis showing any abnormality of cerebral spinal fluid like Hydrocephalus (accumulation of cerebrospinal fluid)?	1/154 (1)	1-b	(Trans)glycosidases	*GBA, 1:155208421_G/A-AA (0.0%)
				FAD/NAD(P)-binding domain	FMO2, 1:171174531_A/G-AG (15.97%)
GO:0032782 bile acid secretion	Intrahepatic cholestasis: impairs the release of a digestive fluid called bile from liver cells. As a result, bile builds up in the liver, impairing liver function If you're pregnant women, have you been diagnosed with Intrahepatic cholestasis?	1/52 (1)	2-b	P-loop containing nucleoside triphosphate hydrolases	*ABCB6, 2:220075193_G/C-CC (0.0%)<br>*TAP2, 6:32797849_C/G-GG (0.0%)<br>*ABCC12, 16:48149369_G/C-CC (0.9%)<br>*ABCC11, 16:48242379_G/A-AG (2.46%)<br>ABCC2, 10:101611294_G/A-AA (6.79%)<br>ABCC12, 16:48122582_G/A-GG (40.88%)
				ABC transporter transmembrane region	*ABCB6, 2:220079136_A/T-TT (0.58%)<br>*ABCC11, 16:48250140_G/C-CC (0.04%)<br>*ABCC2, 10:101595996_T/A-AA (3.73%)
				PDZ domain-like	PDZRN4, 12:41946539_G/A-AA (16.67%)<br>DEPTOR, 8:121061879_G/A-AA (29.95%)
MP:0002896 abnormal bone mineralization	Have you had a bone mineral density test that showed abnormal deposition of bone mineral or Have you been diagnosed with bone mineralization disorders like osteomalacia (softening of the bones), osteogenesis Imperfecta (skeletal dysplasias)?	3/62 (1)	1-b	EGF/Laminin	*MASP2, 1:11105542_C/T-CT (0.32%)
				Metalloproteases ("zincins") catalytic domain	ADAM7, 8:24339679_G/A-AA (26.14%)
DO:DOID:2789 parasitic protozoa infectious disease	Have you ever underwent ova & parasite (O&P) test or stool test showing the presence of Giardia Lamblia (parasite) and been diagnosed with Giardia infection (parasitic infection) which is associated with symptoms like watery diarrhoea, greasy stools, fatigue, cramps, abdominal pain, bloating, malnutrition and indigestion?	1/49 (1)	2-b	Riboflavin synthase domain-like	*OXNAD1, 3:16327909_C/T-TT (17.73%)
				Flavoproteins	*NQO1, 16:69748869_G/A-AG (2.14%)<br>MTRR, 5:7870973_A/G-GG (36.42%)<br>NOS2, 17:26096597_G/A-AG (16.53%)
				Toll/Interleukin receptor TIR domain	TIRAP, 11:126162843_C/T-CT (8.59%)
				Cytokine	IL1F10, 2:113832312_T/C-TT (47.7%)<br>IL36A, 2:113763575_A/G-AA (15.93%)

Ontology term	Question	Num answers: Yes/Total (Predicted yes)	Types predicted	Superfamily/Pfam Domain	Gene, chr:pos_ref/alt(GRCh37)-Genotype (MAF in 1000G) *high-scoring variant
GO:2000611 positive regulation of thyroid hormone generation	Have you ever had blood test and been diagnosed with Graves' disease which is characterized by anxiety and irritability, weight loss, despite normal eating habits, erectile dysfunction, bulging eyes, rapid or irregular heartbeat?	3/143 (1)	2-b	Trypsin-like serine proteases	*TMPRSS4, 11:117982495_T/G-TT (2.08%)<br>PRSS38, 1:228033197_A/G-AA (20.27%)<br>CELA3B, 1:22307538_C/T-CT (10.66%)<br>CELA2B, 1:15813843_G/A-AG (1.12%)<br>OVCH1, 12:29642573_A/C-AA (42.25%)
				SRCR-like	*TMPRSS2, 21:42852497_C/T-TT (26.14%)
MP:0010402 ventricular septal defect	Ventricular septum is the wall dividing the lower chambers of the heart. Have you undergone Echocardiography showing any defect in ventricular septum?	1/47 (1)	1-a	SMAD MH1 domain	*SMAD9, 13:37453762_A/G-AG (0.1%)
GO:1900076 regulation of cellular response to insulin stimulus	Have you or anyone in your family ever had glucose tolerance test showing insulin resistance which may indicate Rabson–Mendenhall syndrome or do you have a family history of reporting symptoms like growth abnormalities of the head, face and nails, along with skin hyperpigmentation (acanthosis nigricans)?	2/75 (1)	2-b	C2 domain (Calcium/lipid-binding domain CaLB)	*SYT8, 11:1858262_C/T-TT (18.55%)
				(Phosphotyrosine protein) phosphatases II	*PALD1, 10:72289778_C/T-TT (12.8%)<br>PTPN18, 2:131127651_A/G-AG (0.38%)<br>DUSP23, 1:159752066_G/A-AG (6.45%)
				Protein kinase-like (PK-like)	TRIB3, 20:368905_A/G-GG (19.91%)
				"Winged helix" DNA-binding domain	HIST1H1T, 6:26108168_G/A-AG (21.7%)
				Cystatin/monellin	CSTL1, 20:23424638_C/T-CT (12.74%)
				Insulin-like	INSL5, 1:67266756_T/A-AT (12.88%)
				SH3-domain	PLCG1, 20:39797465_T/C-CT (26.72%)

1b. Same as 1a. Listing the ontology terms whose percentage of positive answers in people with low outlier scores is below 5% (even though overall positive rate is above 5%).

Ontology term	Question	Num answers: Yes/Total (Predicted yes)	Types predicted	Superfamily/Pfam Domain	Gene, chr:pos_ref/alt(GRCh37)-Genotype (MAF in 1000G) *high-scoring variant
HP:0012732 Anorectal anomaly	Have you undergone endoscopy analysis showing any abnormalities in anus and rectum such as proctitis (inflammation of anus and the lining of the rectum), anal cancer?	3/31 (3)	2-b<br>1-b<br>2-b	CalX-like<br><br>ATPase domain of HSP90 chaperone/DNA topoisomerase II/histidine kinase	*FREM2, 13:39343822_C/T-TT (14.18%)<br>*ADGRV1, 5:89914925_T/G-GT (1.64%)<br>ADGRV1, 5:89979589_G/A-GG (19.63%)<br>ADGRV1, 5:89943571_G/T-GG (18.97%)<br>*ADGRV1, 5:89914925_T/G-GG (1.64%)<br>FREM2, 13:39422624_C/T-CT (10.96%)<br><br>SACS, 13:23930055_A/T-AT (8.65%)
HP:0002164 Nail dysplasia	Dysplastic nails are a cutaneous condition, and may be a subtle finding of ridging, flaking, or poor growth of the nails, or more diffuse with nearly complete loss of nails. Have you noticed anyone in your family have dysplastic nails such as broad nail, split nail or Have you been diagnosed with Nail–patella syndrome (poorly developed nails)?	7/50 (4)	2-b<br>1-b<br>2-b<br>2-b	Keratin type II head	*KRT5, 12:52913668_C/T-TT (2.66%)<br>KRT75, 12:52827818_T/C-TT (38.72%)<br>KRT78, 12:53242440_A/G-AA (48.46%)<br>KRT78, 12:53242440_A/G-GG (48.46%)<br>KRT4, 12:53207628_G/A-AA (22.17%)<br>*KRT5, 12:52913668_C/T-TC (2.66%)<br>KRT2, 12:53045626_T/C-CC (37.56%)
GO:0072512 trivalent inorganic cation transport	Have you ever had blood test showing elevated serum liver enzymes or elevation of the transferrin saturation which may indicate haemochromatosis (iron overload disorder) which cause fatigue, discomfort, joint and bone pain, abnormal heart rhythm, liver disease ?	4/31 (3)	1-b<br>1-b<br>1-b	Ferric reductase like transmembrane component	*STEAP1B, 7:22532986_C/A-AC (6.03%)<br>NOX5, 15:69328226_C/T-TT (27.12%)
GO:0001824 blastocyst development	Have you undergone ultrasound showing congenital abnormalities like phocomelia (malformations of the arms and legs), atrial septal defect (ASD) (heart disease) ?	2/35 (2)	2-b<br>2-b	TPR-like<br><br>lambda repressor-like DNA-binding domains	*IFIT2, 10:91065949_A/C-AC (5.17%)<br>IFT88, 13:21205192_G/A-GG (23.52%)<br>IFIT2, 10:91066769_C/A-AC (12.12%)<br>HELZ, 17:65212042_C/T-CT (19.59%)<br>*TTC29, 4:147788709_C/T-TT (40.84%)<br>SMYD4, 17:1704296_C/A-AA (28.73%)<br><br>POU5F1B, 8:128428638_G/A-AA (37.34%)
GO:0036089 cleavage furrow formation	Have you undergone blood test or electromyogram (ECM) showing muscle spasm or muscle cramp which is characterized by muscle pain, muscle stiffness ?	3/45 (2)	2-b<br>2-b	SH3-domain<br><br>P-loop containing nucleoside triphosphate hydrolases<br><br>FYVE/PHD zinc finger	*PLCG1, 20:39797465_T/C-TT (26.72%)<br><br>RERGL, 12:18234256_T/C-CC (8.29%)<br>RAB40A, X:102755551_T/A-AT (9.59%)<br><br>SP110, 2:231036860_C/T-TC (3.95%)

Ontology term	Question	Num answers: Yes/Total (Predicted yes)	Types predicted	Superfamily/Pfam Domain	Gene, chr:pos_ref/alt(GRCh37)-Genotype (MAF in 1000G) *high-scoring variant
HP:0002861 Melanoma	Melanoma is a type of skin cancer that develops from the pigment-containing cells known as melanocytes. Have you been diagnosed with melanoma?	2/38 (2)	1-b<br>2-b	Cysteine proteinases	*NAT1, 8:18079746_C/T-TC (0.12%)<br>TGM4, 3:44948479_C/T-CC (32.55%)<br>NAT2, 8:18257854_T/C-CC (29.27%)<br>*NAT1, 8:18079746_C/T-CT (0.12%)<br>CAPN12, 19:39229089_A/G-AG (11.32%)<br>TGM3, 20:2297790_G/A-AG (11.18%)<br>CTSS, 1:150727539_G/A-AA (38.24%)<br>CAPN11, 6:44141088_G/A-AG (14.18%)<br>CTSB, 8:11710888_G/C-GG (39.6%)
HP:0000494 Downslanted palpebral fissures	Have you noticed downslanted opening between the eye lids in any of your family members?	3/58 (3)	1-a<br>1-a<br>1-a	GTPase activation domain GAP	*NF1, 17:29586054_T/C-TC (0.0%)<br>*IQGAP2, 5:75964507_C/T-CT (0.54%)
GO:0051385 response to mineralocorticoid	Increased mineralocorticoid activity leads to hypervolemia, hypertension, and hypokalemia Have you had a blood test that showing too much fluid (hypervolemia) or very low level of potassium (hypokalemia) and diagnosed with hypertension?	10/191 (1)	1-a	Leucine zipper domain	*FOS, 14:75747512_G/C-CC (0.26%)
HP:0000639 Nystagmus	Do you or anyone your family have rapid and uncontrolled eye movement with vision impairment or have you been diagnosed with Nystagmus?	2/24 (2)	2-b<br>2-b	ARM repeat	*AP1G2, 14:24033027_G/A-AA (4.35%)<br>AP4B1, 1:114438951_A/G-AG (37.7%)<br>AP4E1, 15:51217361_T/C-TT (44.09%)
				Periplasmic binding protein-like I	*GUCY2F, X:108708552_A/G-GG (16.18%)<br>GUCY2F, X:108708552_A/G-G (16.18%)
				WD40 repeat-like	WDR92, 2:68364478_T/C-CC (17.73%)<br>SEC31B, 10:102269085_C/A-AA (19.41%)<br>CDC20B, 5:54410099_G/A-AG (16.95%)<br>SEC31B, 10:102269206_A/G-GG (18.53%)<br>GNB1L, 22:19808769_C/T-CT (14.4%)<br>LRBA, 4:151199080_G/A-AA (14.22%)<br>LRBA, 4:151207127_C/T-TT (16.21%)<br>WDR31, 9:116085423_G/A-AG (6.23%)<br>DMXL2, 15:51829812_G/A-AA (12.84%)<br>WDR4, 21:44273858_G/A-AA (34.34%)<br>SPAG16, 2:214727221_A/C-CC (38.42%)<br>WDR55, 5:140048544_C/T-CT (20.05%)
				Voltage-gated potassium channels	CATSPER4, 1:26520292_G/T-GT (2.34%)<br>TPCN2, 11:68846399_A/T-AT (9.96%)
				Tetraspanin	TSPAN18, 11:44940828_G/A-AA (34.7%)
				MIR domain	POMT1, 9:134390870_C/A-AC (1.26%)
