# Supplementary material for: Hypothesis-free phenotype prediction within a genetics-first framework
Source: Nat Commun. 2023 Feb 17;14:919. doi: 10.1038/s41467-023-36634-6 (PMC9938118; doi:10.1038/s41467-023-36634-6)
Supplement: Supplementary file 3 — Supplementary Data 1-5 [file 41467_2023_36634_MOESM3_ESM.zip › Supplementary Data/Supplementary Data 2.pdf]

Supplimentary Table 2. List of predicted phenotypes in DDD cohort for each person.

For each person with a matched prediction, showing the clinically annotated ontology terms, the predictions that matched annotations, types of matched predictions, and contributing variants. Diagnostic status: 0 if no DNM or diagnosis is found, 1 if DNM is found but not interpreted with DDG2P, 2 if a genetic diagnosis have been provided with DDG2P.

Diagnostic status	Clinical annotation	Exactly/closely matched prediction	Types of matched prediction	Superfamily/Pfam Domain	Gene, chr:pos_ref/alt(GRCh37)-Genotype (MAF in 1000G) *high-scoring variant
2	HP:0001331 Absent septum pellucidum	HP:0001331 Absent septum pellucidum	1-b	HMG-box	*TOX3, 16:52479976_T/A-TA (0.0%)<br>*SOX2, 3:181430372_T/C-TC (0.0%)
1	HP:0000834 Abnormality of the adrenal glands	HP:0011733 Abnormality of adrenal physiology<br>HP:0000834 Abnormality of the adrenal glands<br>HP:0000859 Hyperaldosteronism<br>HP:0002717 Adrenal overactivity<br>HP:0011732 Abnormality of adrenal morphology<br>HP:0008221 Adrenal hyperplasia	2-b<br>2-b<br>2-b<br>2-b<br>2-b<br>2-b	Cytochrome P450	*CYP2A6, 19:41352842_G/A-GA (0.02%)<br>*CYP4F11, 19:16035680_G/A-AA (0.1%)<br>*CYP4B1, 1:47282772_C/T-TT (15.83%)<br>*CYP4F2, 19:15996828_G/C-CC (0.42%)<br>*CYP4B1, 1:47280884_C/T-TT (13.5%)<br>*CYP2C18, 10:96493058_C/T-TT (21.88%)<br>CYP2C8, 10:96818119_G/C-GC (1.66%)<br>CYP2C19, 10:96534922_G/C-GC (0.9%)<br>CYP2D6, 22:42523943_A/G-AA (35.92%)<br>CYP2A7, 19:41383217_T/C-CC (18.57%)<br>CYP2W1, 7:1028448_C/T-CT (18.25%)
				Voltage-gated potassium channels	*KCNG2, 18:77659260_G/T-GT (0.0%)<br>*KCNG1, 20:49626146_G/T-GT (0.0%)
				E set domains	PKHD1L1, 8:110394748_C/G-CG (0.0%)<br>FLNC, 7:128488734_G/A-GA (6.25%)
				Tyrosine-dependent oxidoreductases	BLVRB, 19:40964395_C/T-CT (0.18%)<br>HSD17B13, 4:88231429_G/A-GA (2.22%)<br>NMRAL1, 16:4519439_G/A-GA (3.21%)
0	HP:0004912 Hypophosphatemic rickets	HP:0100529 Abnormality of phosphate homeostasis	2-b	G proteins	*IFT27, 22:37158995_G/A-AA (1.12%)<br>*ARL5C, 17:37321347_G/A-GA (1.96%)<br>*RAB33B, 4:140375527_G/T-GT (0.0%)<br>*RAB13, 1:153958633_C/A-CA (0.0%)<br>MX1, 21:42815731_G/A-GA (0.78%)<br>RHOF, 12:122218783_T/G-TG (1.98%)
				Alkaline phosphatase-like	PIGN, 18:59821843_T/C-TC (6.53%)
0	HP:0000494 Downslanted palpebral fissures	HP:0000494 Downslanted palpebral fissures	1-b	p120GAP domain-like	*RASAL3, 19:15565594_C/T-CT (0.0%)<br>*RASAL3, 19:15565646_G/A-GA (1.0%)
1	HP:0002365 Hypoplasia of the brainstem	HP:0002363 Abnormality of brainstem morphology	2-b	Nucleotide-diphospho-sugar transferases	*GALNT8, 12:4855365_G/T-GT (0.3%)<br>*A3GALT2, 1:33772792_C/T-CT (0.18%)<br>GALNT12, 9:101570336_A/T-AT (2.86%)<br>GALNTL5, 7:151684256_A/G-GG (22.06%)
1	HP:0000369 Low-set ears	HP:0000369 Low-set ears	1-b	Ras family	*ERAS, X:48687676_G/T-GT (0.0%)<br>AGAP2, 12:58128378_C/G-CG (0.16%)<br>RERGL, 12:18234256_T/C-TC (8.29%)
				XPF/Rad1/Mus81 nuclease	MUS81, 11:65631361_C/T-CT (1.24%)
0	HP:0010627 Anterior pituitary hypoplasia	HP:0010625 Anterior pituitary dysgenesis	1-a	Homeodomain-like	*TIGD6, 5:149375886_C/T-CT (0.14%)

Diagnostic status	Clinical annotation	Exactly/closely matched prediction	Types of matched prediction	Superfamily/Pfam Domain	Gene, chr:pos_ref/alt(GRCh37)-Genotype (MAF in 1000G)<br>*high-scoring variant
0	HP:0010627<br>Anterior pituitary hypoplasia<br>HP:0000830<br>Anterior hypopituitarism	HP:0000839<br>Pituitary dwarfism<br>HP:0040086<br>Abnormal prolactin level<br>HP:0040075<br>Hypopituitarism<br>HP:0000830<br>Anterior hypopituitarism<br>HP:0000824<br>Growth hormone deficiency	1-a<br>1-a<br>1-a<br>1-a<br>1-a	Homeodomain-like	*TIGD6, 5:149375886_C/T-CT (0.14%)
2	HP:0008034<br>Abnormal iris pigmentation	HP:0001088<br>Brushfield spots	1-b	RING/U-box	*RNF216, 7:5754803_A/G-AG (0.0%)<br>TRIM15, 6:30131711_G/C-GC (1.34%)<br>TRIM10, 6:30128442_C/T-CT (9.09%)
2	HP:0008034<br>Abnormal iris pigmentation<br>HP:0000262<br>Turricephaly	HP:0000262<br>Turricephaly<br>HP:0000244<br>Brachyturricephaly	1-b<br>1-b	RING/U-box	*RNF216, 7:5754803_A/G-AG (0.0%)<br>TRIM15, 6:30131711_G/C-GC (1.34%)<br>TRIM10, 6:30128442_C/T-CT (9.09%)
2	HP:0008034<br>Abnormal iris pigmentation<br>HP:0000262<br>Turricephaly<br>HP:0000248<br>Brachycephaly	HP:0000248<br>Brachycephaly	1-b	RING/U-box<br>TB module/8-cys domain	*RNF216, 7:5754803_A/G-AG (0.0%)<br>TRIM15, 6:30131711_G/C-GC (1.34%)<br>TRIM10, 6:30128442_C/T-CT (9.09%)<br>LTBP3, 11:65319751_G/A-GA (6.29%)
2	HP:0000657<br>Oculomotor apraxia	HP:0002186<br>Apraxia	1-a	Extended AAA-ATPase domain	*SPATA5L1, 15:45695445_C/G-GG (1.0%)
2	HP:0000964<br>Eczema	HP:0001051<br>Seborrheic dermatitis<br>HP:0007569<br>Generalized seborrheic dermatitis<br>HP:0000964<br>Eczema	2-b<br>2-b<br>2-b	Low-density lipoprotein receptor domain class A<br>LDL receptor-like module<br>Thrombospondin type 1 domain	*TMPRSS6, 22:37467003_C/A-CA (0.0%)<br>*MALRD1, 10:19780551_A/G-AG (0.36%)<br>*MALRD1, 10:19676553_A/G-GG (12.82%)<br>ADAMTS14, 10:72500763_T/C-TT (20.01%)
0	HP:0008850<br>Severe postnatal growth retardation	HP:0008850<br>Severe postnatal growth retardation	1-b	Homeodomain-like	*TIGD5, 8:144680308_C/T-CT (0.0%)<br>*ALX3, 1:110607238_G/A-GA (0.0%)<br>NKX2-6, 8:23560484_G/T-GT (1.06%)
2	HP:0002373<br>Febrile seizures	HP:0002373<br>Febrile seizures	1-b	Voltage-gated potassium channels<br>Neurotransmitter-gated ion-channel transmembrane pore	*KCNK1, 1:233767033_G/T-TT (1.18%)<br>CHRNA9, 4:40356041_C/T-CT (14.18%)
0	HP:0000535<br>Sparse eyebrow	HP:0100840<br>Aplasia/Hypoplasia of the eyebrow<br>HP:0000535<br>Sparse eyebrow	2-b<br>2-b	Acyl-CoA N-acyltransferases (Nat)<br>Keratin type II head	*NAA10, X:153197863_G/A-GA (0.0%)<br>SAT2, 17:7529902_G/A-GA (7.63%)<br>KRT79, 12:53227788_C/T-CT (1.1%)<br>KRT75, 12:52827818_T/C-TT (38.72%)<br>KRT78, 12:53242440_A/G-GG (48.46%)<br>KRT4, 12:53207628_G/A-AA (22.16%)<br>KRT3, 12:53189696_C/G-CG (13.84%)

Diagnostic status	Clinical annotation	Exactly/closely matched prediction	Types of matched prediction	Superfamily/Pfam Domain	Gene, chr:pos_ref/alt(GRCh37)-Genotype (MAF in 1000G) *high-scoring variant
1	HP:0000766 Abnormality of the sternum	HP:0000767 Pectus excavatum	2-b	Collagen triple helix repeat (20 copies)	*COL9A2, 1:40768374_C/A-CA (0.0%)<br>*MBL2, 10:54531235_C/T-TT (12.2%)<br>COL11A2, 6:33141280_G/A-GA (10.22%)<br>COL27A1, 9:117002765_G/A-GA (0.02%)
				G proteins	*DRG2, 17:18003923_C/T-CT (0.14%)<br>ENSG00000180574, 12:10659228_A/T-TT (16.65%)<br>ARL5C, 17:37319065_C/T-CT (13.22%)<br>LRRK2, 12:40702911_G/A-GA (10.04%)
				Fibrillar collagen C-terminal domain	COL2A1, 12:48367976_C/T-CT (17.09%)
2	HP:0000494 Downslanted palpebral fissures	HP:0000494 Downslanted palpebral fissures	1-b	p120GAP domain-like	*RASAL3, 19:15565646_G/A-GA (1.0%)
				BRK domain-like	*CHD6, 20:40040870_G/A-GA (1.2%)
2	HP:0000160 Narrow mouth	HP:0000160 Narrow mouth	2-b	Collagen triple helix repeat (20 copies)	*MBL2, 10:54531235_C/T-TT (12.2%)<br>*COL23A1, 5:177683905_G/A-GA (5.33%)<br>*COL19A1, 6:70851789_A/G-AG (0.22%)<br>COL4A3, 2:228110691_C/A-CA (0.26%)<br>COL4A3, 2:228131169_A/G-AG (11.14%)<br>COL18A1, 21:46911188_C/G-CG (6.89%)
1	HP:0009765 Low hanging columella	HP:0009929 Abnormality of the columella	1-a	HMG-box	*BBX, 3:107435532_C/T-CT (0.3%)
2	HP:0001680 Coarctation of aorta	HP:0011004 Abnormality of the systemic arterial tree	2-b	4-helical cytokines	*IFNA14, 9:21239447_G/A-AA (0.42%)<br>*IFNA16, 9:21216934_G/A-AA (2.6%)<br>IFNA16, 9:21216910_A/G-GG (2.6%)<br>IFNA16, 9:21217111_A/T-TT (2.6%)<br>IFNA16, 9:21217166_T/C-CC (1.64%)<br>IFNA16, 9:21217022_C/G-GG (2.6%)<br>IFNA16, 9:21216908_C/G-GG (2.6%)
2	HP:0000733 Stereotypic behavior	HP:0000733 Stereotypic behavior	1-b	Calcium-dependent phosphotriesterase	*APMAP, 20:24944483_G/A-GA (0.4%)
				Serum paraoxonase/arylesterase 1 PON1	PON2, 7:95034775_G/C-CC (28.33%)
0	HP:0001258 Spastic paraplegia	HP:0010549 Weakness due to upper motor neuron dysfunction	2-b	Complement control module/SCR domain	*CFHR2, 1:196918741_G/A-AA (1.52%)<br>*CR1L, 1:207867854_G/A-GA (3.93%)<br>*CR1L, 1:207851554_C/T-CT (3.97%)<br>CR1L, 1:207868022_G/T-GT (3.95%)<br>CR1L, 1:207857254_A/G-GG (16.11%)<br>CR1, 1:207755348_C/T-CT (0.42%)<br>CR1L, 1:207890892_G/A-GA (2.98%)
				Ion transport protein	*SCN5A, 3:38629033_G/A-GA (0.0%)<br>ENSG00000262304, 17:3477195_G/A-GA (0.04%)<br>CACNA1H, 16:1245509_G/C-GC (0.5%)<br>SCN7A, 2:167298015_C/T-CT (0.64%)<br>TPCN2, 11:68846399_A/T-AT (9.96%)
2	HP:0005795: Short tapering fingers	HP:0100807 Long fingers	2-b	Calcium-binding EGF domain	*FBN3, 19:8150247_C/A-CA (0.0%)<br>*FBN3, 19:8197930_C/T-CT (0.0%)<br>*FBN3, 19:8176890_C/G-CG (0.38%)<br>SCUBE1, 22:43623395_C/G-CC (26.34%)<br>ADGRE1, 19:6901923_G/T-GT (0.4%)
0	HP:0001680 Coarctation of aorta	HP:0011004 Abnormality of the systemic arterial tree	1-a	4-helical cytokines	*IFNA10, 9:21206605_C/G-CG (0.46%)

Diagnostic status	Clinical annotation	Exactly/closely matched prediction	Types of matched prediction	Superfamily/Pfam Domain	Gene, chr:pos_ref/alt(GRCh37)-Genotype (MAF in 1000G) *high-scoring variant
0	HP:0000733 Stereotypic behavior	HP:0000733 Stereotypic behavior	1-a	Calcium-dependent phosphotriesterase	*RGN, X:46943961_A/G-G (0.0%)
2	HP:0000684 Delayed eruption of teeth	HP:0000684 Delayed eruption of teeth	1-b	PX domain	*SNX21, 20:44469366_A/C-AC (0.08%)<br>SNX15, 11:64802388_A/C-AC (3.69%)
1	HP:0000517 Abnormality of the lens	HP:0010696 Polar cataract	2-b	Crystallins/Ca-binding development proteins	*CRYBG3, 3:97634465_A/G-AG (0.36%)<br>CRYBB3, 22:25599849_G/A-GA (2.52%)
				Tetraspanin	TSPAN10, 17:79612633_T/C-TC (8.49%)
0	HP:0000762 Decreased nerve conduction velocity	HP:0040129 Abnormal nerve conduction velocity<br>HP:0003134 Abnormality of peripheral nerve conduction<br>HP:0045010 Abnormality of peripheral nerves	2-b<br>2-b<br>2-b	Serum paraoxonase/arylesterase 1 PON1	*PON1, 7:94931564_C/T-CT (0.0%)<br>PON1, 7:94940880_A/C-AC (0.26%)<br>PON2, 7:95034775_G/C-CC (28.33%)
0	HP:0001270 Motor delay	HP:0001270 Motor delay	2-b	KRAB domain (Kruppel-associated box)	*ZNF674, X:46388286_A/C-C (0.0%)<br>ZNF461, 19:37147421_A/G-AG (0.0%)<br>ZNF135, 19:58572979_G/A-GA (21.75%)<br>ZNF254, 19:24289358_G/A-GA (0.0%)<br>ZNF543, 19:57838018_C/G-GG (45.21%)
				Connexin	*GJE1, 6:142454996_C/T-CT (0.34%)<br>*GJA4, 1:35260208_C/T-CT (0.42%)
				MIR domain	*SDF2L1, 22:21997333_C/A-CA (0.0%)
				Sodium ion transport-associated	SCN9A, 2:167099158_A/G-AG (11.28%)
1	HP:0001627 Abnormality of the heart	HP:0001671 Abnormality of the cardiac septa	2-b	EGF-type module	*DLK2, 6:43419744_C/T-CT (0.0%)
				TGF-beta propeptide	BMP10, 2:69093439_G/C-GC (0.2%)
				Calcium-binding EGF domain	SCUBE1, 22:43623395_C/G-CC (26.34%)<br>FBN2, 5:127609633_G/A-GA (7.57%)
				TB module/8-cys domain	FSTL3, 19:677797_G/A-GA (0.0%)
				Ankyrin repeats (3 copies)	KANK4, 1:62728784_A/G-AA (28.41%)<br>BARD1, 2:215632255_C/T-TT (36.62%)<br>KANK4, 1:62728838_T/C-TT (28.37%)<br>ESPN, 1:6500343_C/T-CT (0.0%)

Diagnostic status	Clinical annotation	Exactly/closely matched prediction	Types of matched prediction	Superfamily/Pfam Domain	Gene, chr:pos_ref/alt(GRCh37)-Genotype (MAF in 1000G) *high-scoring variant
0	HP:0000864 Abnormality of the hypothalamus-pituitary axis	HP:0010625 Anterior pituitary dysgenesis<br>HP:0000839 Pituitary dwarfism<br>HP:0040086 Abnormal prolactin level<br>HP:0040075 Hypopituitarism<br>HP:0000830 Anterior hypopituitarism<br>HP:0011751 Abnormality of the posterior pituitary<br>HP:0011753 Posterior pituitary dysgenesis<br>HP:0000824 Growth hormone deficiency<br>HP:0000871 Panhypopituitarism	1-b<br>1-b<br>1-b<br>1-b<br>1-b<br>1-b<br>1-b<br>1-b<br>1-b	Homeodomain-like	*HOXB13, 17:46804358_G/A-GA (0.0%)<br>ISX, 22:35478529_G/A-GA (3.08%)<br>HOXD12, 2:176965306_A/C-AC (0.0%)
				Homeodomain	*HOXB13, 17:46804358_G/A-GA (0.0%)<br>ISX, 22:35478529_G/A-GA (3.08%)<br>HOXD12, 2:176965306_A/C-AC (0.0%)
1	HP:0000098 Tall stature	HP:0000098 Tall stature<br>HP:0001548 Overgrowth	2-b<br>1-b	Growth factor receptor domain	*NOTCH2, 1:120512157_C/T-CT (0.0%)
				vWA-like	ITIH5, 10:7622009_G/A-GA (5.93%)<br>COL6A3, 2:238296306_G/C-GC (0.18%)<br>CLCA1, 1:86952324_A/G-AG (11.3%)<br>SVEP1, 9:113312231_G/C-GC (1.32%)<br>MATN4, 20:43926573_C/T-TT (21.87%)
				EGF/Laminin	USH2A, 1:216462662_T/A-TA (3.45%)
1	HP:0000098 Tall stature<br>HP:0000256 Macrocephaly	HP:0040194 Increased head circumference<br>HP:0000256 Macrocephaly	1-b<br>1-b	Growth factor receptor domain	*NOTCH2, 1:120512157_C/T-CT (0.0%)
				EGF/Laminin	USH2A, 1:216462662_T/A-TA (3.45%)
0	HP:0000494 Downslanted palpebral fissures	HP:0000494 Downslanted palpebral fissures	1-b	p120GAP domain-like	*RASAL3, 19:15565646_G/A-GA (1.0%)
				BRK domain-like	*CHD6, 20:40040870_G/A-GA (1.2%)
2	HP:0000490 Deeply set eye	HP:0000490 Deeply set eye	1-b	Periplasmic binding protein-like II	*MELTF, 3:196742363_C/T-CT (0.0%)<br>TF, 3:133494354_C/T-TT (15.63%)
0	HP:0002558 Supernumerary nipples	HP:0002558 Supernumerary nipple	2-b	EGF-type module	*MASP2, 1:11105542_C/T-CT (0.32%)<br>CRTAC1, 10:99640120_C/T-CT (0.7%)<br>ADGRE1, 19:6896544_G/T-GT (0.0%)<br>CRB2, 9:126128253_G/C-CC (31.59%)<br>HEG1, 3:124728626_A/G-AA (37.18%)
1	HP:0005795: Short tapering fingers	HP:0001182 Tapered finger	1-a	FHA domain	*APLF, 2:68717350_A/T-AT (0.08%)
2	HP:0001182 Tapered finger	HP:0100807 Long fingers	1-b	Calcium-binding EGF domain	*MASP2, 1:11105542_C/T-CT (0.32%)<br>CD93, 20:23065584_C/T-CT (0.28%)<br>FBN2, 5:127685135_C/T-CC (26.9%)
0	HP:0002376 Developmental regression	HP:0002376 Developmental regression	1-a	IF2B-like	*MRI1, 19:13876824_T/C-TC (0.0%)

Diagnostic status	Clinical annotation	Exactly/closely matched prediction	Types of matched prediction	Superfamily/Pfam Domain	Gene, chr:pos_ref/alt(GRCh37)-Genotype (MAF in 1000G)<br>*high-scoring variant
0	HP:0001518<br>Small for gestational age	HP:0001518<br>Small for gestational age	1-a	RuvA domain 2-like	*FAAP24, 19:33467575_C/T-CT (0.14%)
0	HP:0001518<br>Small for gestational age<br>HP:0004325<br>Decreased body weight	HP:0001518<br>Small for gestational age	1-a	RuvA domain 2-like	*FAAP24, 19:33467575_C/T-CT (0.14%)
1	HP:0011304<br>Broad thumb	HP:0011304<br>Broad thumb	2-b	Calponin-homology domain CH-domain<br>KRAB domain (Kruppel-associated box)	*CNN2, 19:1036542_C/T-CT (0.0%)<br>*ZNF44, 19:12385848_C/G-CG (1.54%)<br>*ZNF764, 16:30569349_C/T-CT (0.92%)<br>ZNF30, 19:35422808_C/T-TT (28.81%)<br>ZKSCAN2, 16:25263278_G/A-AA (33.47%)
2	HP:0001511<br>Intrauterine growth retardation	HP:0001511<br>Intrauterine growth retardation	1-b	Helicase conserved C-terminal domain<br>Restriction endonuclease-like	*DDX56, 7:44609495_G/C-GC (0.0%)<br>ERCC6L2, 9:98691137_T/C-TC (22.7%)<br>*MUS81, 11:65631974_C/G-CG (0.0%)
2	HP:0000776<br>Congenital diaphragmatic hernia	HP:0000775<br>Abnormality of the diaphragm	1-b	HMG-box	*SP100, 2:231379832_C/T-TT (0.0%)<br>*HMGB4, 1:34329932_A/T-AT (0.0%)
1	HP:0001629<br>Ventricular septal defect	HP:0001629<br>Ventricular septal defect<br>HP:0010438<br>Abnormality of the ventricular septum<br>HP:0001713<br>Abnormality of cardiac ventricle	2-b<br>2-b<br>2-b	EGF-type module<br>Ankyrin repeats (3 copies)<br>Calcium-binding EGF domain<br>SET domain<br>Myosin rod fragments<br>LIM domain<br>Glucocorticoid receptor-like (DNA-binding domain)	*NOTCH4, 6:32171629_C/A-CA (0.0%)<br>*FAT2, 5:150891772_C/G-GG (3.33%)<br>KANK1, 9:742394_C/G-CG (0.0%)<br>ASB15, 7:123256427_C/T-CC (22.06%)<br>FBN2, 5:127685135_C/T-CC (26.9%)<br>SMYD1, 2:88387557_A/C-AC (13.66%)<br>*KRT27, 17:38936587_C/T-CT (0.0%)<br>*LPXN, 11:58317528_C/T-CT (0.0%)<br>*ZYX, 7:143085968_C/T-CT (0.66%)<br>NR1H4, 12:100926308_T/C-TC (0.16%)
1	HP:0003189<br>Long nose	HP:0003189<br>Long nose	1-a	HMG-box	*SOX5, 12:23689589_C/G-CG (0.0%)
2	HP:0000407<br>Sensorineural hearing impairment	HP:0000399<br>Prelingual sensorineural hearing impairment<br>HP:0008625<br>Severe sensorineural hearing impairment<br>HP:0011476<br>Profound sensorineural hearing impairment	1-b<br>1-b<br>1-a	Serine protease inhibitors<br>Second domain of FERM	*OTOG, 11:17655400_T/C-TC (0.0%)<br>MYO7B, 2:128393390_G/A-GA (0.54%)
1	HP:0000431<br>Wide nasal bridge	HP:0000431<br>Wide nasal bridge	1-b	A DNA-binding domain in eukaryotic transcription factors<br>Clathrin adaptor core protein	*MAFK, 7:1579709_C/T-CT (0.0%)<br>AP3D1, 19:2138704_T/C-TC (0.0%)

Diagnostic status	Clinical annotation	Exactly/closely matched prediction	Types of matched prediction	Superfamily/Pfam Domain	Gene, chr:pos_ref/alt(GRCh37)-Genotype (MAF in 1000G) *high-scoring variant
1	HP:0000154 Wide mouth	HP:0000154 Wide mouth	1-a	Clathrin coat assembly domain	*AP1S3, 2:224642493_G/A-GA (0.24%)
1	HP:0000322 Short philtrum	HP:0000322 Short philtrum	1-a	Clathrin coat assembly domain	*AP1S3, 2:224642493_G/A-GA (0.24%)
2	HP:0000322 Short philtrum	HP:0000322 Short philtrum	1-a	Clathrin coat assembly domain	*AP1S3, 2:224642493_G/A-GA (0.24%)
1	HP:0001332 Dystonia	HP:0004373 Focal dystonia<br>HP:0012179 Craniofacial dystonia<br>HP:0000473 Torticollis<br>HP:0001332 Dystonia	2-b<br>2-b<br>2-b<br>2-b	Collagen triple helix repeat (20 copies)	*COL7A1, 3:48604374_C/A-CA (0.0%)<br>*MBL2, 10:54531235_C/T-TT (12.2%)<br>*COL4A1, 13:110866346_G/A-GA (0.18%)<br>*COL23A1, 5:177683905_G/A-GA (5.33%)<br>C1QTNF6, 22:37578652_G/A-GA (12.8%)<br>COL18A1, 21:46911188_C/G-CG (6.89%)<br>COL22A1, 8:139701209_G/T-TT (29.17%)
				vWA-like	*MATN1, 1:31188804_G/A-GA (0.0%)<br>VWA2, 10:116045796_G/A-GA (0.66%)<br>SEC23B, 20:18505200_G/T-GT (0.88%)<br>COL6A2, 21:47545768_G/A-AA (39.4%)
2	HP:0002059 Cerebral atrophy	HP:0002120 Cerebral cortical atrophy	2-b	Serum paraoxonase/arylesterase 1 PON1	*PON1, 7:94940880_A/C-AC (0.26%)<br>PON2, 7:95034775_G/C-CC (28.33%)
				Calcium-dependent phosphotriesterase	APMAP, 20:24952147_G/A-GA (2.96%)
2	HP:0000032 Abnormality of male external genitalia	HP:0000028 Cryptorchidism	1-a	Extended AAA-ATPase domain	*MCM2, 3:127335756_G/A-GA (0.0%)
