# Supplementary material for: Hypothesis-free phenotype prediction within a genetics-first framework
Source: Nat Commun. 2023 Feb 17;14:919. doi: 10.1038/s41467-023-36634-6 (PMC9938118; doi:10.1038/s41467-023-36634-6)
Supplement: Supplementary file 4 — Reporting Summary [file 41467_2023_36634_MOESM4_ESM.pdf]

nature portfolio

Corresponding author(s): Julian Gough

Last updated by author(s): Nov 26, 2022

Reporting Summary

Nature Portfolio wishes to improve the reproducibility of the work that we publish. This form provides structure for consistency and transparency in reporting. For further information on Nature Portfolio policies, see our Editorial Policies and the Editorial Policy Checklist.

Statistics

For all statistical analyses, confirm that the following items are present in the figure legend, table legend, main text, or Methods section.

n/a	Confirmed	
☐	☒	The exact sample size (*n*) for each experimental group/condition, given as a discrete number and unit of measurement
☐	☒	A statement on whether measurements were taken from distinct samples or whether the same sample was measured repeatedly
☐	☒	The statistical test(s) used AND whether they are one- or two-sided<br>*Only common tests should be described solely by name; describe more complex techniques in the Methods section.*
☐	☒	A description of all covariates tested
☐	☒	A description of any assumptions or corrections, such as tests of normality and adjustment for multiple comparisons
☐	☒	A full description of the statistical parameters including central tendency (e.g. means) or other basic estimates (e.g. regression coefficient) AND variation (e.g. standard deviation) or associated estimates of uncertainty (e.g. confidence intervals)
☐	☒	For null hypothesis testing, the test statistic (e.g. *F*, *t*, *r*) with confidence intervals, effect sizes, degrees of freedom and *P* value noted<br>*Give P values as exact values whenever suitable.*
☒	☐	For Bayesian analysis, information on the choice of priors and Markov chain Monte Carlo settings
☒	☐	For hierarchical and complex designs, identification of the appropriate level for tests and full reporting of outcomes
☐	☒	Estimates of effect sizes (e.g. Cohen's *d*, Pearson's *r*), indicating how they were calculated

Our web collection on statistics for biologists contains articles on many of the points above.

Software and code

Policy information about availability of computer code

Data collection	Open Source software GenomePrep (https://supfam.mrc-lmb.cam.ac.uk/GenomePrep) was used as part of the data collection pipeline. A custom website was also used for data collection (https://supfam.mrc-lmb.cam.ac.uk/SUPERFAMILY/23andMe/)
Data analysis	The Python packages SciPy v1.7.1, NumPy v1.18.3 were used in some statistical data analysis. The Plink1.9 software package was used to calculate ethnic diversity of one of the cohorts, and R v3.6.2 was used for some of the graphical visualisation. PERL packages PDL v2.018_01 and Statistics 1.6611 were used for covariate analysis, as well as R package glm v0.9.9.8. The VEP v108 tool was used to derive protein-coding changes. IMPUTE2 with VCFtools v0.1.16 and BCFtools v1.15 was used during imputation.

For manuscripts utilizing custom algorithms or software that are central to the research but not yet described in published literature, software must be made available to editors and reviewers. We strongly encourage code deposition in a community repository (e.g. GitHub). See the Nature Portfolio guidelines for submitting code & software for further information.

Data

Policy information about availability of data

All manuscripts must include a data availability statement. This statement should provide the following information, where applicable:

- Accession codes, unique identifiers, or web links for publicly available datasets
- A description of any restrictions on data availability
- For clinical datasets or third party data, please ensure that the statement adheres to our policy

The predictions generated as part of the study are available in the supplementary information files. Additionally we make them available in an interactive, searchable format via a webpage at https://supfam.org/nomaly. The DTC cohort data may not be made publicly available because participants are not consented for this, but on application to the corresponding author, efforts will be made to satisfy any reasonable request that falls within the constraints of ethical approval

granted for the project. The database of questions corresponding to 5,857 ontology terms is also available via the resources webpage (above) and may be a valuable resource for other studies. The similarity mapping, by information content, of all HPO terms that are close to the HPO terms used in clinical annotations by DDD are also made available on the resources webpage. All data on the resources webpage are also available for download in JSON format. Third party datasets include data from: 1000G project, DDD, HipSci, GO, HPO, DO, MeSH, MPO. Availability, means to access these datasets, and links to accession codes are provided in the data availability statement.

Field-specific reporting

Please select the one below that is the best fit for your research. If you are not sure, read the appropriate sections before making your selection.

☒ Life sciences ☐ Behavioural & social sciences ☐ Ecological, evolutionary & environmental sciences

For a reference copy of the document with all sections, see nature.com/documents/nr-reporting-summary-flat.pdf

Life sciences study design

All studies must disclose on these points even when the disclosure is negative.

Sample size	Sample sizes are direct integer numbers of participants. The sample sizes of the DDD and HipSci cohorts were dictated by the data in those projects, and the sample size for the DTC cohort was determined by the number of participants we were able to recruit.
Data exclusions	As fully described in the paper, for some of the results presented, phenotypes with a high rate of self-identification were excluded.
Replication	Some of the statistical results are replicated once across both DDD and DTC cohorts and the qualitative result was the same.
Randomization	There was no allocation into groups.
Blinding	Investigators were blinded to participant identity and ethnicity, however there are no relevant groupings for blinding.

Reporting for specific materials, systems and methods

We require information from authors about some types of materials, experimental systems and methods used in many studies. Here, indicate whether each material, system or method listed is relevant to your study. If you are not sure if a list item applies to your research, read the appropriate section before selecting a response.

Materials & experimental systems

n/a	Involved in the study	
☒	☐	Antibodies
☐	☒	Eukaryotic cell lines
☒	☐	Palaeontology and archaeology
☒	☐	Animals and other organisms
☐	☒	Human research participants
☐	☒	Clinical data
☒	☐	Dual use research of concern

Methods

n/a	Involved in the study	
☒	☐	ChIP-seq
☒	☐	Flow cytometry
☒	☐	MRI-based neuroimaging

Eukaryotic cell lines

Policy information about cell lines

Cell line source(s)	The HipSci collection of cell lines include a large number from phenotypically 'healthy' donors, with no diagnosed genetic disease. Cambridge BioResource contributed the tissue samples for this cohort to the HipSci project. For detailed information about each line, please visithttps://www.hipsci.org/cells.
Authentication	These lines are all banked at ECACC (European Collection of Authenticated Cell Cultures) – the authors acknowledge Wellcome Sanger Institute as the source of human induced pluripotent cell line which was generated under the Human Induced Pluripotent Stem Cell Initiative funded by a grant from the Wellcome Trust and Medical Research Council, supported by the Wellcome Trust (WT098051) and the NIHR/Wellcome Trust Clinical Research Facility, and acknowledges Life Science Technologies Corporation as the provider of Cytotune.
Mycoplasma contamination	Cells were screened for the presence of mycoplasma using a standard PCR kit (Promocell, PCR Mycoplasma Test Kit I/C, PK-CA91-1096), the PCR products were loaded onto gel and found to be negative when observed after electrophoresis. All these cell lines had tested negative before in house, or as part of HipSci QC at the Wellcome Sanger Institute within before shipping.
Commonly misidentified lines (See ICLAC register)	N/A

Human research participants

Policy information about studies involving human research participants

Population characteristics	Population characteristics are plotted in Supplementary Figure 1. However this was not used, and also age and gender were not used. Characteristics of the DDD cohort are covered by the paper referenced for their study.
Recruitment	For the DTC cohort participants were recruited online, restricted to those with access to direct-to-consumer genetics data. This cohort is heavily biased to particular demographics. Recruitment of the DDD cohort is covered by the paper referenced for their study.
Ethics oversight	The DTC cohort study was granted ethics approval by the University of Bristol, via the Faculty of Engineering Research Ethics Committee with approval ID number 539500 (project ID 361 and amendment 2322).

Note that full information on the approval of the study protocol must also be provided in the manuscript.

Clinical data

Policy information about clinical studies

All manuscripts should comply with the ICMJE guidelines for publication of clinical research and a completed CONSORT checklist must be included with all submissions.

Clinical trial registration	n/a
Study protocol	Data taken from DDD (referenced in the paper)
Data collection	Data taken from DDD (referenced in the paper)
Outcomes	n/a
